# Comparing Bona Fide Psychotherapies of Depression in Adults with Two Meta-Analytical Approaches

**DOI:** 10.1371/journal.pone.0068135

**Published:** 2013-06-28

**Authors:** Sarah R. Braun, Bettina Gregor, Ulrich S. Tran

**Affiliations:** University of Vienna, Vienna, Austria; University of Melbourne, Australia

## Abstract

**Objective:**

Despite numerous investigations, the question whether all bona fide treatments of depression are equally efficacious in adults has not been sufficiently answered.

**Method:**

We applied two different meta-analytical techniques (conventional meta-analysis and mixed treatment comparisons). Overall, 53 studies with 3,965 patients, which directly compared two or more bona fide psychotherapies in a randomized trial, were included. Meta-analyses were conducted regarding five different types of outcome measures. Additionally, the influence of possible moderators was examined.

**Results:**

Direct comparisons of cognitive behavior therapy, behavior activation therapy, psychodynamic therapy, interpersonal therapy, and supportive therapies versus all other respective treatments indicated that at the end of treatment all treatments but supportive therapies were equally efficacious whereas there was some evidence that supportive therapies were somewhat less efficacious than all other treatments according to patient self-ratings and clinical significance. At follow-up no significant differences were present. Age, gender, comorbid mental disorders, and length of therapy session were found to moderate efficacy. Cognitive behavior therapy was superior in studies where therapy sessions lasted 90 minutes or longer, behavior activation therapy was more efficacious when therapy sessions lasted less than 90 minutes. Mixed treatment comparisons indicated no statistically significant differences in treatment efficacy but some interesting trends.

**Conclusions:**

This study suggests that there might be differential effects of bona fide psychotherapies which should be examined in detail.

## Introduction

In psychotherapy research, the assumption that all psychotherapies are equally efficacious is known as the dodo bird verdict [1]. In the field of depressive disorders, meta-analyses have come to different conclusions regarding its verity: While several meta-analyses promoted the superiority of cognitive therapy for depression [2]–[4], other studies maintained that interpersonal therapy may be the most efficacious treatment [5], [6]. Still, other meta-analyses presented evidence that all psychotherapies are equally efficacious [7], [8].

The above mentioned meta-analyses of Tolin [4] and Wampold et al. [8] introduced an important aspect: They included only psychotherapies that were considered ‘bona fide’. The bona fide definition demands a high standard for treatments to be included in comparisons. It requires that treatments are therapeutically intended and are based on a clear rationale. Minimal and “intent-to-fail” psychological interventions that are occasionally implemented to control for common factors are thus rigorously excluded from comparisons.

The comparable efficacy of bona fide psychotherapies of depression reported by Wampold et al. [8] fitted with considerations of the contextual model of psychotherapy [9], which states that unifying common factors like the therapeutic relationship, a clear rationale of the treatment as well as the patient’s and therapist’s belief in the treatment mediate clinical change. If common factors account for clinical change, all types of psychotherapies are supposed to perform with equal efficacy. In contrast, the more recent meta-analysis of Tolin [4] found that cognitive behavior therapy (CBT) may be superior to other bona fide therapies in the treatment of depression, suggesting that specific factors, i.e., specific techniques within CBT may also affect treatment efficacy.

The interpretation of these conflicting findings is complicated by several factors. First, equally efficacious specific techniques of different psychotherapies (i.e., specific factors) may also result in comparable efficacy [10]. This assumption seems especially plausible given numerous studies on the relative efficacy of psychotherapy and pharmacotherapy which were not able to find any relevant difference [7], [11], [12], [13]. Second, therapeutic mechanisms of change in psychotherapy are still not identified empirically [14], occasionally turning the discussion about common and specific factors into an ideological one. Third, study results might be affected by researcher allegiance, i.e., the extent by which researchers are associated with a certain type of therapy. Whether researcher allegiance affects results in trials which evaluate the efficacy of treatments of depressive disorders has been discussed controversially within several meta-analyses [4], [12], [15]. Fifth, meta-analyses [4], [8] may have been overly restricted in their study sample as they included only 10 studies each which actually focused on bona fide treatments of depressive disorders: The meta-analysis of Wampold et al. [8] was based on a re-analysis of a previously investigated study sample [3] that contained only 10 studies comparing exclusively psychotherapies that were considered bona fide. The meta-analysis of Tolin [4] investigated the general efficacy of CBT compared to other bona fide therapies in the treatment of several disorders. While it included 26 studies in total, only 10 studies sprecifically addressed depressive disorders. Moreover, focussing on CBT, the Tolin meta-analysis [4] did not cover studies comparing bona fide psychotherapies that did not include CBT.

Therefore, one objective of the current study was to update the evidence on bona fide psychotherapies for depression for recently published studies but, second, also to clarify and specifically investigate the status of non-CBT bona fide therapies in comparison to CBT. Supportive therapies including those which referred to the work of Carl Rogers were of special interest in this regard. Wampold [9] considered Rogerian therapy as bona fide because it promotes a theory of the therapeutic change. Yet, the Wampold et al. [4] meta-analysis did not include any studies on bona fide supportive therapies at all, while the Tolin meta-analysis [8] included only one in the field of depressive disorders.

Furthermore, we investigated the utility of novel meta-analytical approaches in examining the relative efficacy of different psychotherapeutic approaches. We compared two different meta-analytical approaches: (1) Conventional meta-analysis, comparing selected types of treatments against all others on relative treatment efficacy as it was done in other meta-analyses [4], [5]; and (2) mixed treatment comparisons [16], also known as network meta-analysis. While conventional meta-analysis is restricted to a serial examination of one specified treatment against all others, mixed treatment comparisons are based on a Bayesian approach and allow the simultaneous examination of all treatments against all others, combining both direct and indirect evidence; i.e., if a study sample contains comparisons of treatments A vs. B, and B vs. C, but not of A vs. C, this last comparison may be indirectly obtained in network meta-analysis. Mixed treatment comparisons have been recently developed but are increasingly used in medical research [17]; for a recent example see [18]. To our knowledge, this methodology was only once applied in examinations of the relative efficacy of psychological interventions in coronary heart disease [19], but never in terms of the relative efficacy of different psychotherapies in the treatment of depressive disorders.

A third focus of the current study pertained to identify moderators that may explain the relative efficacy of different psychotherapies. Researcher allegiance may affect differences in treatment efficacy [12], [20]. While adjusting for researcher allegiance alleviated differences between treatments in some analyses [12], [21], it failed to do so in others [4], [14]. It is still not clear how researcher allegiance may be eliminated at all or how well meta-analytical reviews may adjust for it. Yet, in terms of a conservative analysis it should be considered as a possible confounder [22] and was therefore also examined in our study. Furthermore, we investigated different outcomes and also examined the influence of study population, treatment, and study characteristics on outcome [23]. This may be important not only with regard to treatments themselves but also with regard to patient characteristics and their interaction which may influence outcome [24].

## Methods

### Identification and Selection of Studies

**Figure 1:**
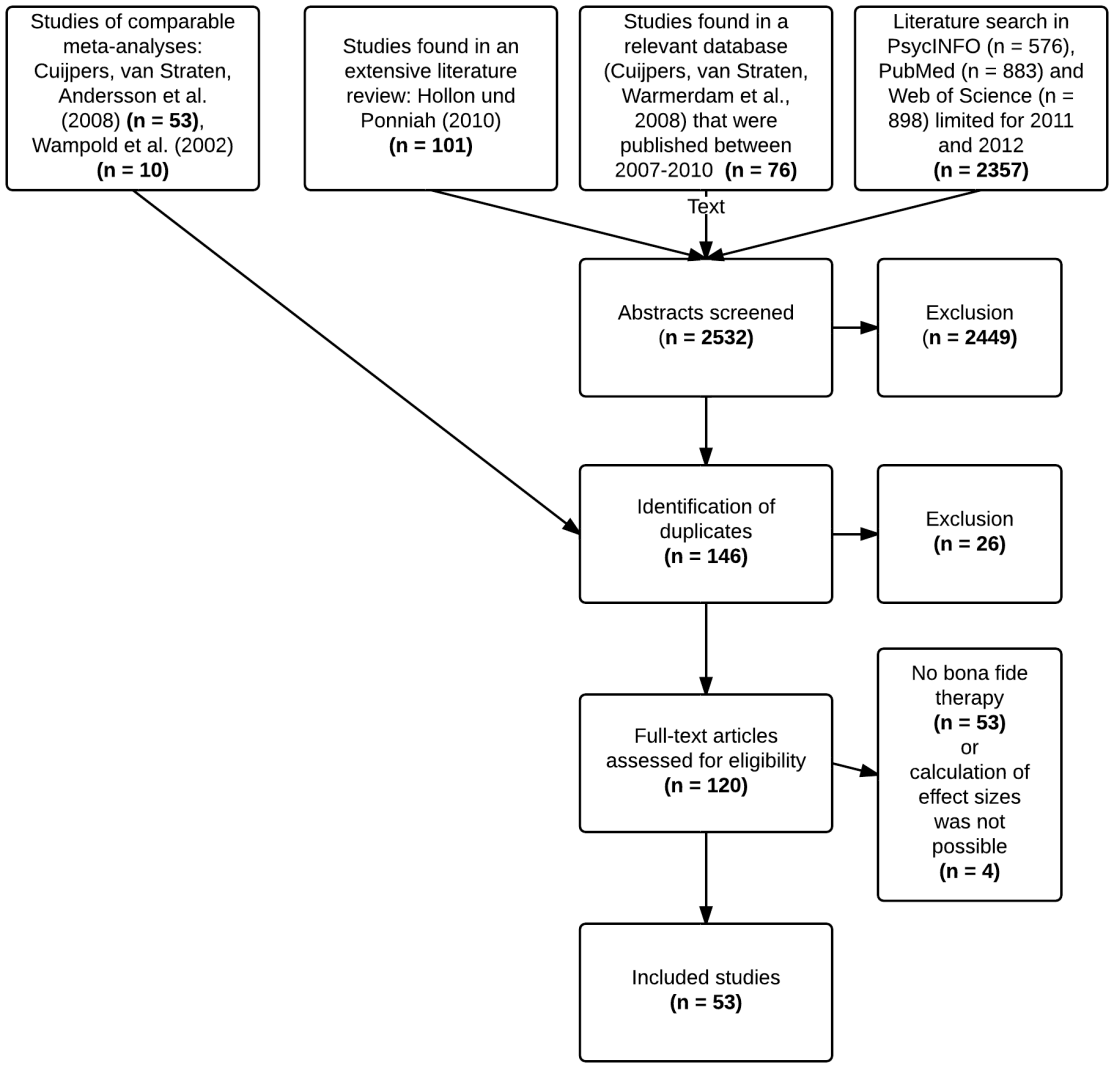
Flow of studies through the selection process.

Details concerning flow of studies through the selection process are shown in Figure 1. Three search strategies were used to identify eligible studies: First, studies included in the meta-analysis of Wampold et al. [8] and studies included in the meta-analysis of Cuijpers et al. [5] were acquired. Second, the abstracts of studies listed in an extensive recent review [25] were screened and studies that were collected in a comprehensive online database [26], which contains articles published up to the end of 2010 and which has also been used for the meta-analysis of Cuijpers et al. [5], were examined. This database has been established by a comprehensive literature search of the major bibliographical databases (PubMed; PsycINFO; Embase; Cochrane Central Register of Controlled Trials) and by examination of the references of 22 prior meta-analyses on the treatment of depression. Third, a literature search was conducted in PsycINFO, MEDLINE (using PubMed) and Web of Science, covering the time span of January 2011 to June 2012 that was not accounted by the other above mentioned sources. We combined key words indicative of psychological treatment (*psychotherapy, interpersonal psychotherapy, psychodynamic psychotherapy, cognitive therapy, behavior therapy, humanistic psychotherapy, brief psychotherapy, experiential psychotherapy, geriatric psychotherapy, analytical psychotherapy, individual psychotherapy, expressive psychotherapy, supportive psychotherapy, adlerian psychotherapy, group psychotherapy, integrative psychotherapy, eclectic psychotherapy*) with key words indicative of depressive disorder (*major depression, endogenous depression, reactive depression, postpartum depression, spreading depression, depression[emotion], recurrent depression, atypical depression, treatment resistant depression*) [5].

For selection of studies, the inclusion criteria of Cuijpers et al. [5] (p. 910) were used: Studies had to compare the efficacy of a psychological treatment for a depressive disorder or an elevated level of depressive symptomatology in adults (all participants in a study had to be 18 years or older) with another psychological treatment in a randomized trial. There were no language restrictions.

In a second step, only those studies were kept in which at least two treatments fulfilled all three criteria of bona fide psychotherapies [8] (pp. 161–162): (I) Therapists needed to be trained in the provided treatment and needed to hold at least a master’s degree or were enrolled in a relevant graduate program (e.g., clinical psychology, counseling psychology, social work). (II) Therapists met face-to-face with the patients and the treatment was individualized for the patients (i.e., no delivery of a standard protocol to each patient). (III) Treatments contained psychologically valid components, fulfilling at least two of the following conditions: Articles contained (a) a citation to an established school or approach to psychotherapy; (b) a description of the therapy that contained a reference to a psychological process (e.g., operant conditioning); (c) a reference to a treatment manual that was used to guide the delivery of the treatment; (d) the identification of active ingredients of the treatment and citations for these ingredients.

In a last step, four studies were excluded in which provided information did not allow the calculation of an effect size. Finally, 53 studies proved to be eligible and were included in the analyses.

### Coding of Studies

According to other meta-analyses in this area [4], [5], the following variables were considered as possible moderators: Study population (presence of a clinical diagnosis, recruitment method, age, gender, comorbid mental disorders, additional intake of psychotropic drugs, type of target group [“purely” depressed patients vs. specific target groups such as patients with a postpartum depression or a specific medical condition], marital status, BDI pretest score), treatment (number and length of therapy sessions, format [individual vs. group], training and supervision of the therapists, whether the same therapists conducted the treatments to be compared, therapists’ adherence to the treatment manual), and study characteristics (randomization [stratified vs. not stratified], study quality, researcher allegiance, type of analysis [intention-to-treat vs. per-protocol], publication year). All included studies were coded by the first author, SRB. BG coded nearly half (24) of these studies independently. Study quality and researcher allegiance was only coded once, by SRB. Agreement for doubly coded variables was good; 93% of the items were coded identically. Ambiguity in the 24 doubly rated studies and uncertainty in ratings of the remaining 29 studies, study quality, and researcher allegiance was resolved by discussion and consensus between SRB and UST.

Study quality was assessed by means of the *risk of bias assessment tool* [27] which allows the judgment of high, low or unclear risk concerning six different types of bias (selection, performance, detection, attrition, reporting, and other bias). Selection bias refers to a random sequence generation as well as allocation concealment, performance bias assesses the blinding of participants and personnel, detection bias evaluates the blinding of outcome assessment, attrition bias refers to incomplete outcome data, reporting bias describes selective reporting and finally, other bias subsumes other sources of bias which were not addressed before.

Researcher allegiance was classified according to Gaffan et al. [14] who distinguished between 3 =  *strong*, 2 =  *moderate*, 1 =  *weak*, and 0 =  *no* allegiance to a certain therapy. For each comparison of two treatments, the difference between the respective allegiance values was calculated resulting in a score of –3 to 3 for each comparison as it was done by [21]. Prevalence of comorbid mental disorders was assessed by determining the percentage of affected participants in each study. In case of multiple comorbid mental disorders, percentage of the most prevalent disorder was used if overall percentage was not reported [28].

### Calculation of Effect Sizes

Five types of outcome measures were computed for each direct comparison of two treatments. This was done in order to take all possible information which was included in the primary studies into account. Furthermore, different types of outcome measures provide the opportunity to analyze whether results for different types of outcome measures pointed into the same direction. Independent effect sizes (Cohen’s *d* which was transformed into Hedges’ *g* accounting for small sample bias) were calculated, first, for patient self-ratings (e.g., Beck Depression Inventory) and, second, for clinician ratings (e.g., Hamilton Depression Rating Scale) of depression severity at posttest and at follow-up. Third, a combined outcome measure was calculated by taking the mean of these two effect sizes [5]. The variance of this combined effect size was computed hypothesizing a correlation of *r* = .40 between the two measures [29]. While previous research suggested that patient self-ratings and clinician ratings are not equivalent measures of outcome, it is currently unclear whether this is due to a more conservative assessment of improvement with the former or a higher sensitivity to change with the latter [30]. Hence, both patient self-ratings and clinician ratings were considered primary outcomes in the present study. Fourth, concerning clinical significance, we calculated the ratio of the odds (*OR*) of having remitted under each type of treatment in comparison and, fifth, the *OR* of drop-out. Number of remitted patients as well as drop-out pertained to the definition used in each respective study.

### Meta-Analyses

Types of treatments were classified according to their naming in the primary studies; mostly the primary studies referred to a manual where the respective term of the treatment could be found.

#### Type I

Conventional meta-analysis proceeds by conducting a separate meta-analysis for each type of treatment against all others [5]. Meta-analyses of this type were conducted at posttest and at follow-up for types of treatments where five or more comparisons were available, following [5]. Types of treatments with less than five comparisons were subsumed under “other therapies” and were not investigated individually. Furthermore, meta-analyses on specific comparisons were conducted for pairs of treatments with five or more comparisons available, following again [5]. Random effects models were used throughout and *Q* and *I* ^2^ statistics were calculated. *Q* is the weighted sum of squares on a standardized scale and was compared with the expected weighted sum of squares which assumes that all studies share a common effect; *I* ^2^ describes the ratio of true heterogeneity to total observed dispersion with a range of 0% to 100%. Variability of effect sizes was assumed small for *I* ^2^ = 25%, moderate for *I* ^2^ = 50%, and high for *I* ^2^ = 75% [31]. Publication bias was assessed by inspecting the funnel plots and by applying the trim-and-fill procedure by [32].

Meta-regression was applied to determine associations between effect sizes and study characteristics. If a categorical variable proved to be influential, subgroup analyses were conducted. The software metafor [33] in R (version 2.14.1) was used for this type of meta-analysis.

#### Type II

Mixed treatment comparisons provide the opportunity to combine direct and indirect comparisons and to rank treatments regarding their efficacy [15]. Mixed treatment comparisons require several assumptions: As all available evidence is included, trials must be comparable in terms of patient samples, outcome measures and other relevant characteristics. To account for similarity, we restricted our study sample to studies in which therapies focused on the treatment of depressive disorders and we included only adult patient samples. Furthermore, we analyzed different outcome measures separately to make results more comparable. Also, consistency assumptions must be met, i.e., direct and indirect comparisons must not be biased and should yield comparable results. To examine consistency, we compared results of direct pairwise comparisons within Type I meta-analysis with the respective results of Type II meta-analysis. Results of meta-regressions in Type I meta-analysis served to indicate further possible confounding. We used mixed treatment comparisons to assess the relative treatment efficacy in terms of patient self-ratings, clinician ratings as well as the clinical significance (i.e., remission) for all types of treatments at posttest. As mixed treatment comparisons are based upon Bayesian modeling, they require the specification of relevant parameters distributions in advance [34]. We used random effects models in which correlations between multiple study arms were also taken into account (see supporting information). For all variables of interest non-informative prior distributions were assumed. After a burn-in of 50,000 simulations, 100,000 simulations were conducted on which the presented results are based on. WinBUGS (version 1.4.3) was used for computations.

A comparison between the meta-analyses of Type I and Type II is displayed in Table 1.

**Table 1:** Comparison between Type I and Type II Meta-Analysis.

Conventional Meta-Analysis (Type I)	Mixed Treatment Comparisons (Type II)
based on the frequentist concept of probability [54]	based on the Bayesian concept of probability [54]
does not require the specification of relevant parameter distributions,parameter distributions (e.g., sampling error and between-study variability)are estimated from the data [55]	requires the specification of relevant parameter distributions in advance [34]
if multiple comparisons are included in a primary study, these are mostlytreated as if they were independent of each other [31]	the correlation between multiple comparisons can be taken into account [56]
it is only possible to determine if a certain treatment is best or worst comparedto all other treatments if all available evidence concerning that specifictreatment is taken into account [31]	it is possible to rank treatments regarding their efficacy by combining direct with indirect evidence [16]

## Results

### Description of Included Studies

Descriptive information on included studies as well as references and information on studies excluded in the last step are available as supplementary material to this article (see Appendices S1 and S2; Tables S1 and S2). Overall, 3,965 patients participated in the 53 studies; studies were published between 1977 and 2012 and 32 (60.4%) of them were conducted in the United States. Patients’ mean age ranged from 19.9 to 79.4 years, 70.6% of patients were female. The diagnosis of a depressive disorder was among the inclusion criteria in 41 (77.4%) studies. Specific target groups, e.g., patients with a comorbid disease or women with a postpartum depression, were addressed in 15 (28.3%) studies. On average, 37.4% of the patients showed a comorbid mental disorder. In 45 (84.9%) studies the BDI or BDI II was used as the main outcome measure. BDI pretest scores averaged at 25.6 with a range from 12.50 to 39.30. All 32 studies that reported an outcome assessment administered by a clinician used some version of the HRDS.

Sixty-nine head-to-head comparisons were reported in the 53 studies. Eight studies compared three treatments each. The studies examined cognitive behavioral therapy (43 comparisons), behavior activation therapy (17 comparisons), psychodynamic therapy (13 comparisons), nondirective supportive therapy (21 comparisons), interpersonal therapy (11 comparisons), problem-solving therapy (5 comparisons), social skills training (4 comparisons), acceptance commitment therapy (2 comparisons), mindfulness-based cognitive therapy (1 comparison) and other treatments which did not match one of the named categories (19 comparisons). Therapies were conducted in 14 sessions that lasted 60 minutes on average. Therapies were carried out in an individual format in 39 (73.6%) studies.

Intention-to-treat analyses were reported in 24 (45.3%) studies. Differences in allegiance were strong (–3, –2, 2, 3) in 13 (18.8%) comparisons and moderate (–1, 1) or nonexistent (0) in 29 (42.0%) and 27 (39.1%) comparisons, respectively.

### Type I Meta-Analyses

For cognitive behavior therapy (CBT), behavior activation therapy (BA), psychodynamic therapy (DYN), interpersonal therapy (IPT) and supportive therapies (SUP), separate meta-analyses were conducted. Additionally, CBT was split into two subtypes, one covering cognitive therapy according to the manual of Beck [35], and the other subsuming all other sorts of CBT. Analogously, SUP was subtyped into those explicitly referring to the work of Carl Rogers (1902–1987), and those that did not. For all other therapies separate meta-analyses could not be conducted because of less than five comparisons available for each outcome measure.

The results of the meta-analyses at posttest are presented in Table 2. Results with regard to individual studies are given in the supplementary material. No strong indication of superiority of any treatment could be found: CBT, BA, DYN, and IPT proved equally efficacious. However, SUP referring to Rogers was less efficacious than the other treatments according to patient self-ratings, while SUP not referring to Rogers was less efficacious than the other treatments according to clinician ratings (Table 2). Considering heterogeneity significant *Q* values were observed in analyses that dealt with CBT, CBT according to Beck, BA, DYN and SUP. *I* ^2^ was low to moderate in all analyses; however, confidence intervals were quite large.

**Table 2:** Meta-Analyses of the Efficacy of Different Types of Treatment at Posttest.

	Patient self-ratings					Clinician ratings			Combined effect size			
Treatments	*k*	*g*	*Q*	*I* ^2^ (%)	*k*	*g*	*Q*	*I* ^2^ (%)	*k*	*g*	*Q*	*I* ^2^ (%)
Cognitive behavior therapy	41	–0.01	55.31	5.33	26	–0.04	45.09*	38.11	24	–0.04	54.72**	60.81
		[–0.10, 0.08]		[0, 68.06]		[–0.20, 0.11]		[12.65, 80.52]		[–0.21, 0.13]		[38.90, 86.03]
according to Beck	36	–0.01	50.78*	14.48	25	–0.01	41.30*	27.48	24	–0.02	51.56**	56.71
		[–0.11, 0.10]		[0, 70.54]		[–0.15, 0.14]		[6.39, 81.39]		[–0.18, 0.14]		[35.50, 86.18]
others	7	–0.02	4.51	0								
		[–0.17, 0.14]		[0, 83.26]								
Behavior activation therapy	16	–0.08	33.02**	52.23	12	0.04	16.68	37.60	11	0.03	21.11**	53.25
		[–0.30, 0.14]		[16.71, 86.82]		[–0.21, 0.30]		[0, 85.60]		[–0.22, 0.28]		[3.96, 90.12]
Psychodynamic therapy	11	0.19	20.20*	43.54	7	0.09	3.50	0	6	0.18	8.31	0
		[–0.01, 0.40]		[0, 90.84]		[–0.14, 0.32]		[0, 77.79]		[–0.02, 0.38]		[0, 95.79]
Interpersonal therapy	10	–0.09	16.07	39.36	9	–0.01	14.39	43.41	8	–0.05	17.59*	61.04
		[–0.30, 0.13]		[0, 87.78]		[–0.23, 0.21]		[0, 87.69]		[–0.29, 0.20]		[9.11, 93.07]
Supportive therapies	17	0.14	20.71	22.49	9	0.17	17.51*	49.59	5	0.22	4.22	7.49
		[0.00, 0.29]		[0, 70.86]		[–0.06, 0.40]		[0, 84.48]		[–0.01, 0.45]		[0, 88.33]
referring to Rogers	9	0.26*	11.12	32.96								
		[0.02, 0.49]		[0, 78.49]								
others	8	0.07	9.04	21.96	5^a^	0.36**	2.67	0				
		[–0.12, 0.27]		[0, 83.22]		[0.15, 0.58]		[0, 87.95]				

*Note. k* = number of comparisons. 95% confidence intervals are provided in brackets. Negative effect sizes indicate that the focus treatment was more efficacious than all the other treatments.

a Excluding multiple comparisons.

* *p*<.05,

** *p*<.01.

After performing a sensitivity analysis by excluding those studies in which specific target groups were addressed, DYN performed significantly worse than all the other therapies concerning patient self-ratings (*k* = 8, *g* = 0.28, 95% CI = [0.05, 0.51], *p* = .019).

To account for the risk of artificial reduction of heterogeneity because of including more than one comparison per study in some cases (for 8 studies overall, two comparisons each entered in the analyses), analyses were repeated, using only the comparison with the largest effect size per study. Heterogeneity (*I* ^2^) increased by 8% on average and aggregated effect sizes did not change substantially. In terms of the above mentioned results there were no multiple comparisons regarding SUP not referring to Rogers (clinician rating, see Table 1), whereas two multiple comparisons regarding SUP referring to Rogers (patient self-rating). After excluding these multiple comparisons effect size increased somewhat (*k* = 7, *g* = 0.28 [0.00, 0.56], *p* = .050).

We tested for publication bias by applying the trim-and-fill procedure. For patient self-ratings, for example, the following numbers of studies were imputed (with changed effect sizes and their respective confidence intervals in parentheses): Four studies to the disadvantage of CBT (*g* = 0.02 [–0.07, 0.11]), one study to the disadvantage of IPT (*g* = –0.05 [–0.26, 0.16]), zero studies for DYN and BA, and five studies to the advantage of SUP (*g* = 0.05 [–0.10, 0.20]). Imputing these studies did not change the results significantly. For SUP referring to Rogers, however, three studies were imputed to its advantage resulting in an effect size in patient self-ratings of *g* = 0.13 [–0.08, 0.35] that was not significant (*p* = .226). Results regarding clinician ratings with regard to SUP not referring to Rogers did not change because no studies were imputed.

#### Clinical significance

Results on differences in remission rates are shown in Table 3. Again, no superiority of CBT, BA, DYN or IPT could be found. Yet again, SUP was somewhat less efficacious than the other treatments. In terms of heterogeneity a significant *Q* value was only obtained for DYN. *I* ^2^ was low to moderate and did not increase much after exclusion of multiple comparisons. Again confidence intervals for *I* ^2^ were quite large.

**Table 3:** Meta-Analyses of the Efficacy of Different Types of Treatment at Posttest: Clinical Significance.

Treatments	*k*	*OR*	*Q*	*I* ^2^ (%)
Cognitive behavior therapy	22	1.02 [0.80, 1.31]	25.43	8.60 [0, 66.81]
according to Beck	19	1.08 [0.84, 1.39]	21.21	3.14 [0, 69.04]
others	5	0.84 [0.54, 1.29]	4.07	0 [0, 93.71]
Behavior activation therapy	11	0.97 [0.62, 1.50]	15.65	35.53[0, 82.70]
Psychodynamic therapy	10	0.87 [0.52, 1.45]	16.98*	47.09 [0, 84.02]
Interpersonal therapy	9	1.35 [0.83, 2.20]	13.93	39.08 [0, 88.46]
Supportive therapies	16	0.65** [0.51, 0.83]	14.92	2.18 [0, 62.29]
referring to Rogers	7	0.61** [0.43, 0.88]	15.65	0 [0, 47.74]
others	9	0.71 [0.46, 1.08]	11.96	34.51 [0, 83.47]

*Note. k* = number of comparisons; *OR* = odds ratio. 95% confidence intervals are provided in brackets. *OR*>1 indicates that the odds of remission in the focus treatment were higher than in all the other treatments.

* *p*<.05,

** *p*<.01.

After performing a sensitivity analysis by excluding those studies in which specific target groups were addressed, analyses regarding SUP suggested that this type of treatment was less efficacious than other treatments (SUP: *k* = 6, *OR* = 0.61 [0.42, 0.89], *p* = .010; SUP referring to Rogers: *k* = 4, *OR* = 0.67 [0.45, 1.00], *p* = .047; SUP not referring to Rogers: *k* = 2, *OR* = 0.37 [0.14, 0.96], *p* = .042).

Adjusting for publication bias, two studies were imputed to the advantage of SUP not referring to Rogers (*OR* = 1.14 [0.34, 3.81]). For SUP referring to Rogers and SUP overall zero studies were imputed.

#### Specific comparisons

Table 4 presents the results of the specific comparisons for pairs of treatments for which at least five comparisons were available. CBT and SUP could also be compared with regard to clinical significance, *k* = 5, *OR* = 1.49 [0.81, 2.73], *p* = .165. Again, effect sizes were small and not significant. *Q* values were only significant for BA; *I* ^2^ was low to moderate with large confidence intervals.

**Table 4:** Meta-Analyses of the Efficacy of Different Types of Treatment at Posttest: Specific Comparisons.

Cognitive behavior	Patient self-ratings					Clinician ratings			Combined effect size			
therapy vs.	*k*	*g*	*Q*	*I* ^2^ (%)	*k*	*g*	*Q*	*I* ^2^ (%)	*k*	*g*	*Q*	*I* ^2^ (%)
Supportive therapies	8	–0.05	6.92	0								
		[–0.21, 0.12]		[0, 83.10]								
Behavior activation therapy	7	–0.06	9.20	31.32	7	–0.25	12.66*	53.33	6	–0.15	12.48*	60.73
		[–0.39, 0.27]		[0, 89.81]		[–0.66, 0.16]		[0, 91.39]		[–0.54, 0.23]		[0, 94.57]
Psychodynamic therapy	6	–0.16	4.05	0								
		[–0.36, 0.04]		[0, 84.30]								
Interpersonal therapy	5	0.08	5.97	35.40	5	0.00	8.87	56.32				
		[–0.16, 0.33]		[0, 91.91]		[–0.30, 0.30]		[0, 95.61]				

*Note. k* = number of comparisons. 95% confidence intervals are provided in brackets. Negative effect sizes (*g*) indicate that the focus treatment was more efficacious than the treatment compared.

* *p*<.05.

#### Differences between treatments at follow-up

No indication of difference between any types of treatment could be found at follow-up (30 studies; detailed results omitted for brevity).

#### Drop-out

Table 5 displays the *OR*s of the respective comparisons. Compared to all other treatments, the chance of completing the treatment was significantly lower for CBT according to Beck, but significantly higher for all other CBTs and IPT. In terms of heterogeneity no significant *Q* values were obtained and *I* ^2^ was low to moderate.

**Table 5:** Meta-Analyses on Differences between Different Types of Treatment: Drop-Out.

Treatments	*k*	*OR*	*Q*	*I* ^2^ (%)
Cognitive behavior therapy	42	0.94 [0.73, 1.19]	43.32	18.61[0, 39.53]
according to Beck	36	0.77* [0.61, 0.97]	24.20	0 [0, 14.19]
others	8	1.95* [1.05, 3.63]	9.96	37.10 [0, 76.39]
Behavior activation therapy	17	0.89 [0.57, 1.39]	19.35	25.57 [0, 65.04]
Psychodynamic therapy	12	0.70 [0.45, 1.09]	13.76	17.50 [0, 75.04]
Interpersonal therapy	11	1.65** [1.13, 2.43]	7.75	0 [0, 70.21]
Supportive therapies	21	0.92 [0.68, 1.24]	23.22	26.59 [0, 57.29]
referring to Rogers	10	0.88 [0.63, 1.23]	4.82	0 [0, 48.19]
others	11	0.95 [0.56, 1.60]	17.91	48.48 [0, 80.20]

*Note. k* = number of comparisons; *OR* = odds ratio. 95% confidence intervals are provided in brackets. *OR*>1 indicates that the odds of completing the focus treatment were higher than for all the other treatments.

* *p*<.05,

** *p*<.01.

### Moderator Analyses (Type I Meta-Analyses)

Associations between effect size and study, patient, and treatment characteristics were investigated with moderator analyses. Associations of the more than 25 coded variables with effect size were examined for types of treatment and outcome measures at posttest where heterogeneity was significant (*Q* tests in Table 2) and where at least five independent studies were available. Variables that were found to decrease heterogeneity significantly (*p*<.05) according to respective *Q* tests were treated as candidate moderators. To control the false discovery rate (FDR; i.e., the probability of false positive discoveries) with regard to these candidate moderators, we applied the Benjamini-Hochberg method [36]. Per type of treatment, the overall FDR was set to 10% each to compensate for the known low power of the *Q* test on which this evaluation was based. Moderators surviving this procedure were checked for confounding with other variables. Respective detailed results (including tests on residual heterogeneity, *QE*, and on heterogeneity explained by the moderator, *QM*, as well as subgroup analyses, where applicable) are reported in the following. Notably, differences in allegiance scores were not related to effect size in any analysis (*p*s ≥.080). Likewise, patient characteristics, like presence of a clinical diagnosis of depression, whether specific target groups were addressed, type of recruitment, and BDI pretest score, did not influence relative efficacy (*p*s ≥.067).

#### Study quality

Study quality could not be investigated in moderator analyses at first, because sample sizes for ratings in each category were mostly too small (*k* ≤3). Therefore, we recoded “unclear risk of bias” into “high risk of bias” assuming that the absence of clear evidence of low risk of bias implied a high risk of bias. The impact of all different types of bias was assessed simultaneously when sample size was large enough. According to patient self-rating, DYN was relatively less efficacious than all other therapies in those studies in which there was a high risk for reporting bias (moderator analysis: *k* = 11; *QE*(9) = 10.61, *p* = .304; *QM*(1) = 8.84, *p* = .003; relative efficacy of DYN in studies with high risk of reporting bias: *k* = 8, *g* = 0.34 [0.15, 0.53], *p*<.001).

#### Participant age and treatment format

**Figure 2:**
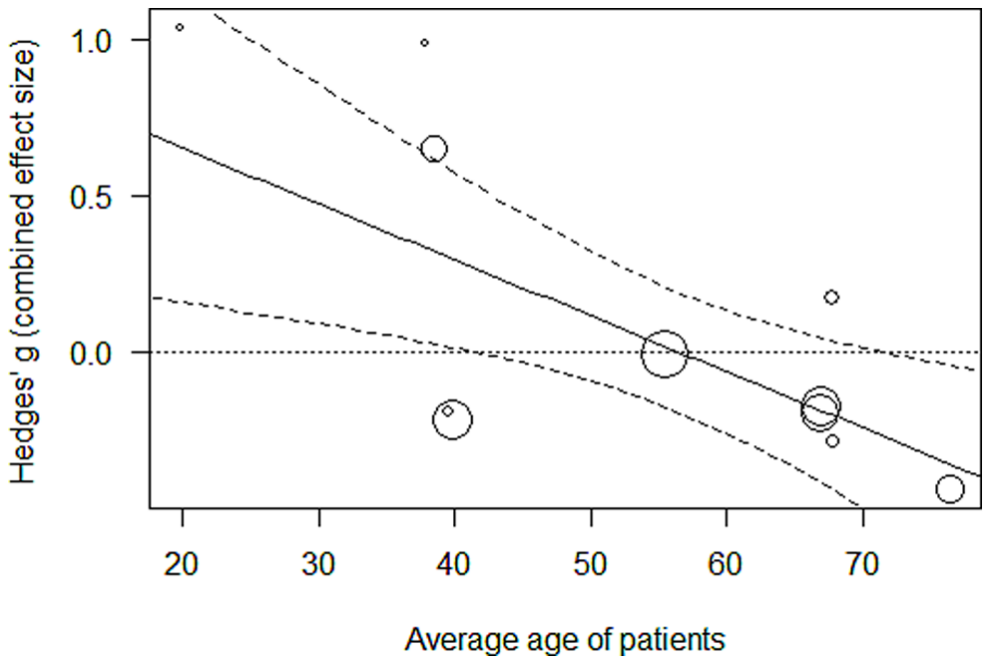
Association between the average age of patients and effect size (combined outcome effect size) with regard to behavior activation therapy. Negative effect sizes indicate that behavior activation therapy was more efficacious than all other treatments.

The relative efficacy of BA according to the combined effect size varied with age in that higher mean age of participants was associated with greater relative efficacy of BA in comparison to other treatments (*k* = 11; *QE*(9) = 12.13, *p* = .206; *QM*(1) = 7.85, *p* = .005; slope estimate = –0.0179 [–0.0305, –0.0054], *p* = .005; the slope estimate indicates how much the effect size increases per one unit of the moderator). Participants over the age of 60 years appeared to benefit more from BA than from other treatments (Figure 2). This effect of participant age could not be observed with regard to patient self-ratings (*k* = 16, *p* = .542). However, we found that format and age were confounded in studies on BA (*r* = –.67, *p* = .013): BA was according to the combined effect size less efficacious than other therapies in a group or couples format (moderator analysis: *k* = 11; *QE*(8) = 2.79, *p* = .947; *QM*(2) = 18.32, *p*<.001; relative efficacy of BA in studies with a group or couples format (*k* = 3, *g* = 0.80 [0.38, 1.21], *p*<.001; in studies with an individual format: *k* = 8, *g* = –0.17 [–0.34, 0.01], *p* = .058). When format and mean age were examined simultaneously, only format appeared to be influential (*k* = 11; *QE*(7) = 2.63, *p* = .917; heterogeneity explained by moderators: *F*(3, 7) = 16.42, *p* = .002; slope estimates: mean age = –0.0029, *p* = .532; group format = 1.0880 [0.4958, 1.6802], *p* = .003; couples format = 0.7531 [0.2798, 1.2264], *p* = .007).

#### Participant sex

CBT was more efficacious according to clinician ratings than other treatments when the proportion of female patients increased (*k* = 26; *QE*(23) = 36.89, *p* = .045; *QM*(2) = 7.26, *p* = .007; slope estimate = –0.0088 [–0.0152, –0.00248], *p* = .005). The proportion of female patients was confounded with publication year (*r* = –.37, *p*<.001), such that the proportion of female patients decreased over time, and was higher in studies where compared treatments were conducted by the same therapists (*r* = .52, *p*<.001). Assessing these variables simultaneously resulted in none of them being influential (*k* = 26, *ps*>.141 for all moderators). However, when proportion of female patients and publication year were examined simultaneously, the former proved to be significantly associated with effect size (*k* = 26; *QE*(23) = 36.24, *p* = .039; *QM*(2) = 8.09, *p* = .018; slope estimates: proportion of female patients = –0.0092 [–0.0156, –0.0029], *p* = .005; publication year: *p* = .488). Whether the same therapists conducted both treatments did not significantly moderate effect size on its own (*k* = 26, *p* = .074).

#### Comorbid disorders

According to the combined effect size, CBT was also more efficacious than other treatments in samples with higher rates of comorbid anxiety disorders (*k* = 5; *QE*(3) = 2.47, *p* = .482; *QM*(1) = 8.87, *p* = .003; slope estimate = –0.0244 [–0.0405, –0.0083], *p* = .003). Rates of comorbid anxiety disorders were highly correlated with rates of personality disorders (*r* = .90, *p*<.001) and the overall proportion of comorbid disorders (*r* = .90, *p* = .037) in these studies. However, comorbid anxiety disorders and comorbid personality disorders could not be evaluated simultaneously (*k* = 4). Furthermore, rates of comorbid anxiety disorders were also fully confounded with checks on the adherence to therapy manuals: They were only reported in studies that also checked adherence.

#### Length of therapy session

Length of therapy session proved to be the most important moderator concerning cognitive behavior therapy (clinician ratings: *k* = 17; *QE*(15) = 27.59, *p* = .024; *QM*(1) = 7.50, *p* = .006; slope estimate = –0.0141 [–0.0242, –0.0040], *p* = .006; combined effect size: *k* = 16; *QE*(14) = 32.71, *p* = .003; *QM*(1) = 8.84, *p* = .003; slope estimate = –0.0147 [–0.0245, –0.0050], *p* = .003) and behavior activation therapy (patient self-ratings: *k* = 13; *QE*(11) = 18.35, *p* = .074; *QM*(1) = 7.79, *p* = .005; slope estimate = 0.0173 [0.0051, 0.0294], *p* = .005; combined effect size: *k* = 7; *QE*(5) = 6.12, *p* = .295; *QM*(1) = 7.02, *p* = .008; slope estimate = 0.0166 [0.0043, 0.0289], *p* = .008). Therefore, further meta-analyses were conducted for all types of outcomes, dividing the study sample into studies with therapy sessions of 90 minutes or longer and studies with therapy session of less than 90 minutes (none of the included treatments had a session length between 60 and 90 minutes).

CBT was more efficacious than other treatments when therapy sessions lasted 90 minutes or longer (Table 6). This was also the true with respect to clinical significance (*k* = 6, *OR* = 2.12 [1.06, 4.25], *p* = .034). In contrast, BA was more efficacious than other treatments when therapy sessions lasted less than 90 minutes. Effects of the length of therapy session disappeared at follow-up (*p*s ≥.307 and *p*s ≥.120 for CBT and BA, respectively). However, it was noted that length of therapy session was highly correlated with format of treatment *(r* = .71, *p*<.001, for CBT and *r* = .72, *p*<.001, for BA) so that longer therapy sessions were conducted in a group format more often. There were only three comparisons in which CBT was conducted in an individual format with therapy sessions longer than 90 minutes. However, concerning self-ratings, CBT was still more efficacious than other treatments in these three comparisons (*g* = –0.48 [–0.93, –0.03], *p* = .035) whereas in those studies (*k* = 15) where individual CBT therapy sessions lasted less than 90 minutes CBT was equally efficacious (*g* = 0.03 [–0.09, 0.15], *p* = .644). For BA there were only two comparisons in which BA was conducted in an individual format and where sessions lasted longer than 90 minutes. Still, considering only the individual format, BA was more efficacious when therapy sessions were shorter than 90 minutes (*k = *7, *g* = –0.37 [–0.67; –0.07], *p* = .017, for self-ratings) and as efficacious as other therapies when only those comparisons were considered in which sessions lasted more than 90 minutes (*k* = 2, *g* = 0.08, [–0.55, 0.70], *p* = .813, for self-ratings).

**Table 6:** Meta-Analyses on the Efficacy of Cognitive Behavior Therapy (CBT) and Behavior Activation Therapy (BA) with Regard to Length of Therapy Session.

	Patient self-ratings				Clinician ratings				Combined effect size			
	*k*	*g*	*Q*	*I* ^2^ (%)	*k*	*g*	*Q*	*I* ^2^ (%)	*k*	*g*	*Q*	*I* ^2^ (%)
Length of therapy session ≥90 minutes												
CBT	11	–0.24*	11.44	2.74	8	–0.31*	7.78	0	7	–0.33*	8.23	12.62
		[–0.46, –0.01]		[0, 75.73]		[–0.59, –0.03]		[0, 82.72]		[–0.59, –0.07]		[0, 88.42]
BA	6	0.26	11.20*	56.49	5	0.42	7.08	44.19				
		[–0.16, 0.69]		[0, 95.14]		[–0.17, 1.02]		[0, 92.81]				
Length of therapy session <90 minutes												
CBT	16	0.03	21.63	0.01	9	0.23	20.85**	61.65	9	0.23	26.44**	73.92
		[–0.08, 0.15]		[0, 84.26]		[–0.06, 0.52]		[17.52, 93.97]		[–0.06, 0.52]		[39.90, 95.24]
BA	7	–0.37*	9.26	39.85								
		[–0.67, –0.07]		[0, 82.30]								

*Note. k* = number of comparisons. 95% confidence intervals are provided in brackets. Negative effect sizes indicate that the focus treatment was more efficacious than all the other treatments.

* *p*<.05,

** *p*<.01.

Furthermore, regarding CBT, length of therapy session was also confounded with type of analysis such as intention-to-treat analysis was performed relatively more often in studies where therapy sessions lasted less than 90 minutes (62%) compared to those where therapy sessions lasted more than 90 minutes (18%). When both moderators, length of therapy session and type of analysis, were examined simultaneously, length of therapy session retained its significance in clinician ratings (*k* = 17, slope estimate = –0.0146 [–0.0290, –0.0002], *p* = .048) and the combined effect size (*k* = 16, slope estimate = –0.0160 [–0.0297, –0.0024], *p* = .024). However, in none of these analyses did type of analysis itself approach significance (*p*s>.612).

### Type II Meta-Analyses

**Figure 3:**
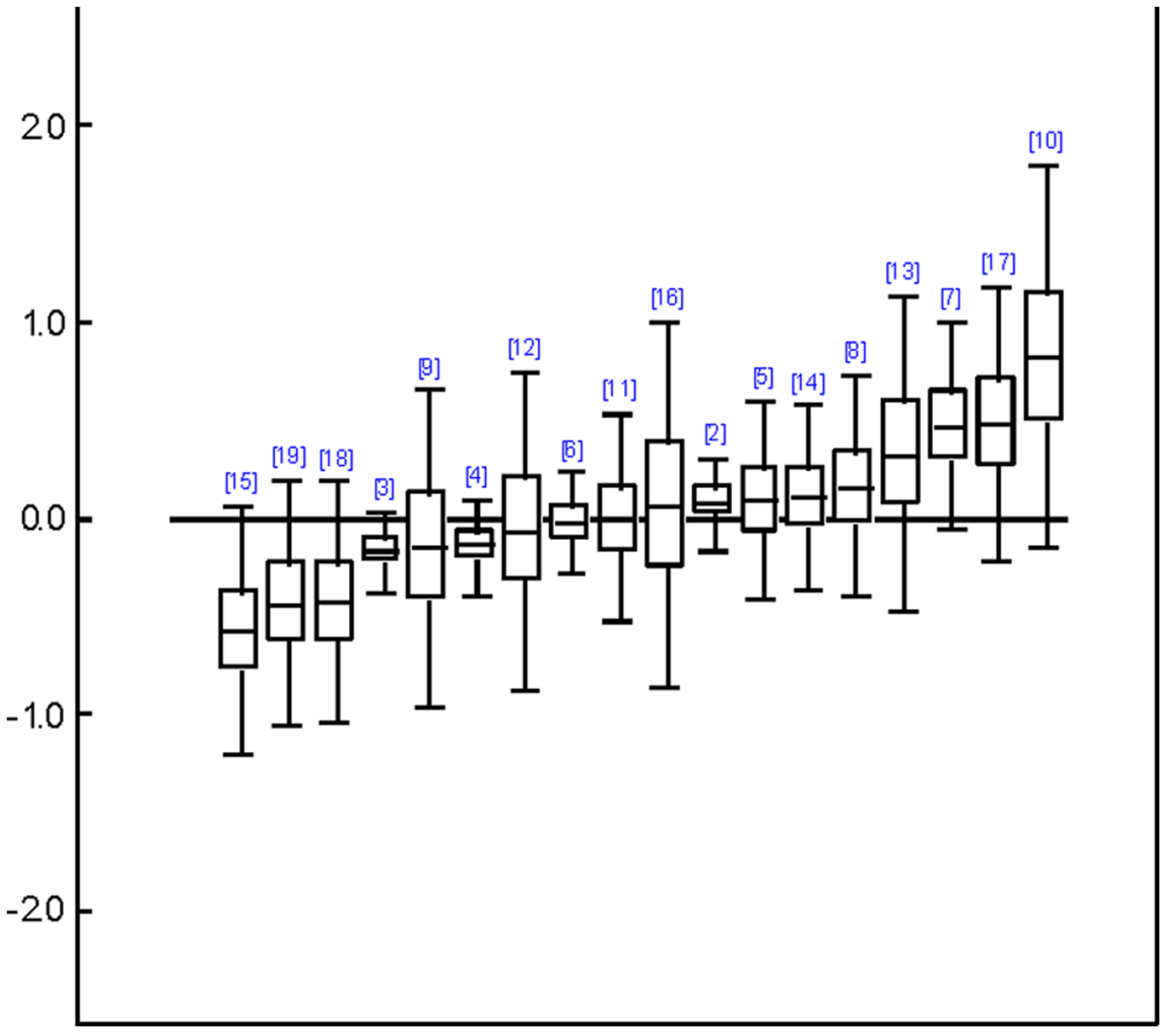
Relative efficacy of different treatments compared to cognitive behavior therapy referring to patient self-ratings. Relative differences of effect sizes are shown with the solid line representing cognitive behavior therapy as reference ( = 1). 2 =  behavior activation therapy; 3 =  supportive therapies; 4 =  psychodynamic therapy; 5 =  problem solving therapy; 6 =  interpersonal therapy; 7 =  social skills training; 8 =  acceptance and commitment therapy; 9 =  mindfulness based cognitive therapy; 10 =  cognitive behavioral analysis system of psychotherapy; 11 =  coping-oriented couples therapy; 12 =  process-experiential therapy; 13 =  self-system therapy; 14 =  emotion focused therapy; 15 =  prescriptive therapy; 16 =  interpersonal process group therapy; 17 =  cognitive hypnosis; 18 =  relational-inside therapy; 19 =  narrative therapy.

**Figure 4:**
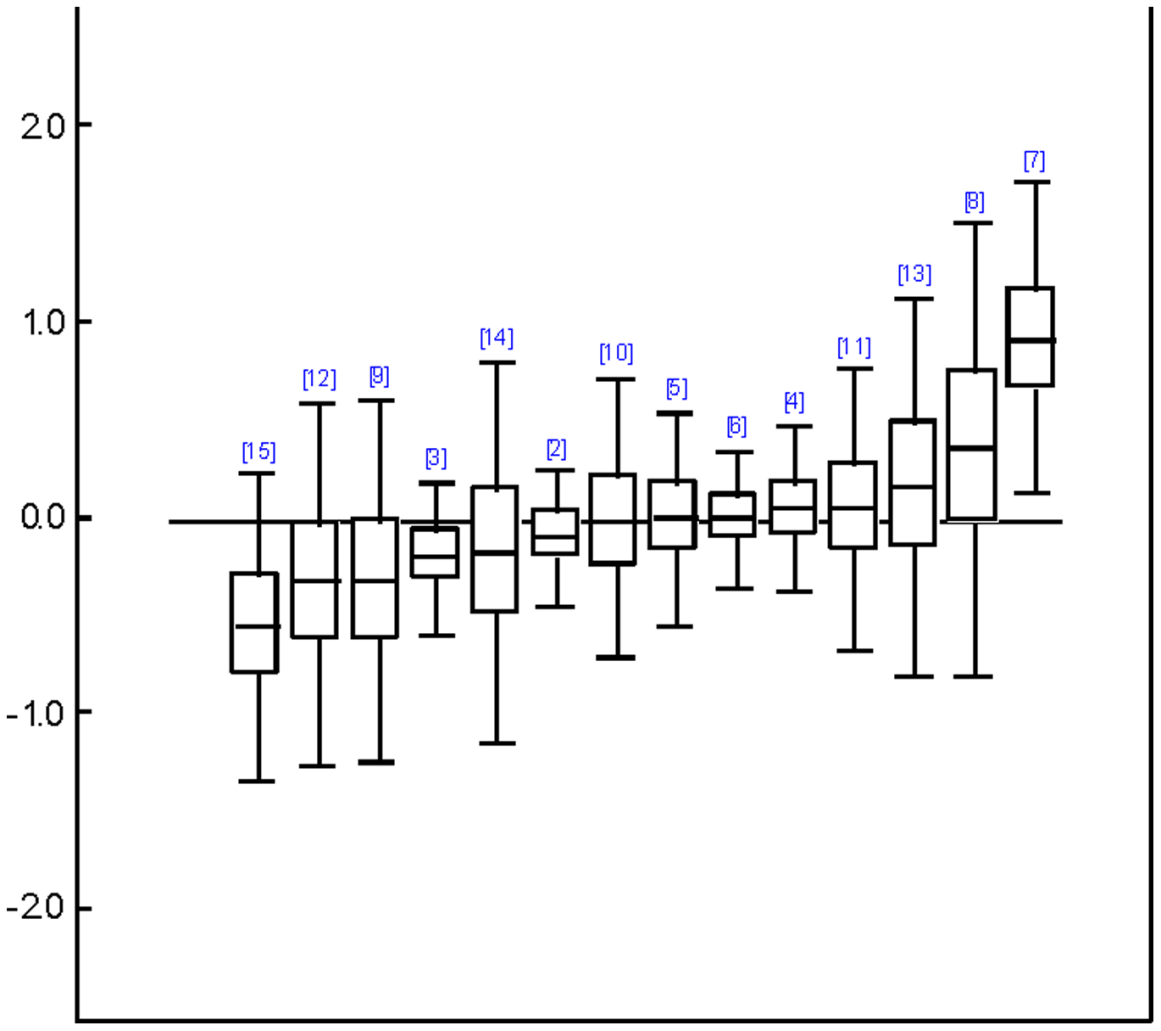
Relative efficacy of different treatments compared to cognitive behavior therapy referring to clinician ratings. Relative differences of effect sizes are shown with the solid line representing cognitive behavior therapy as reference ( = 1). 2 =  behavior activation therapy; 3 =  supportive therapies; 4 =  psychodynamic therapy; 5 =  problem solving therapy; 6 =  interpersonal therapy; 7 =  social skills training; 8 =  acceptance and commitment therapy; 9 =  prescriptive therapy; 10 =  cognitive behavioral analysis system of psychotherapy; 11 =  coping-oriented couples therapy; 12 =  narrative therapy; 13 =  self-system therapy; 14 =  emotion focused therapy; 15 =  relational-insight therapy.

**Figure 5:**
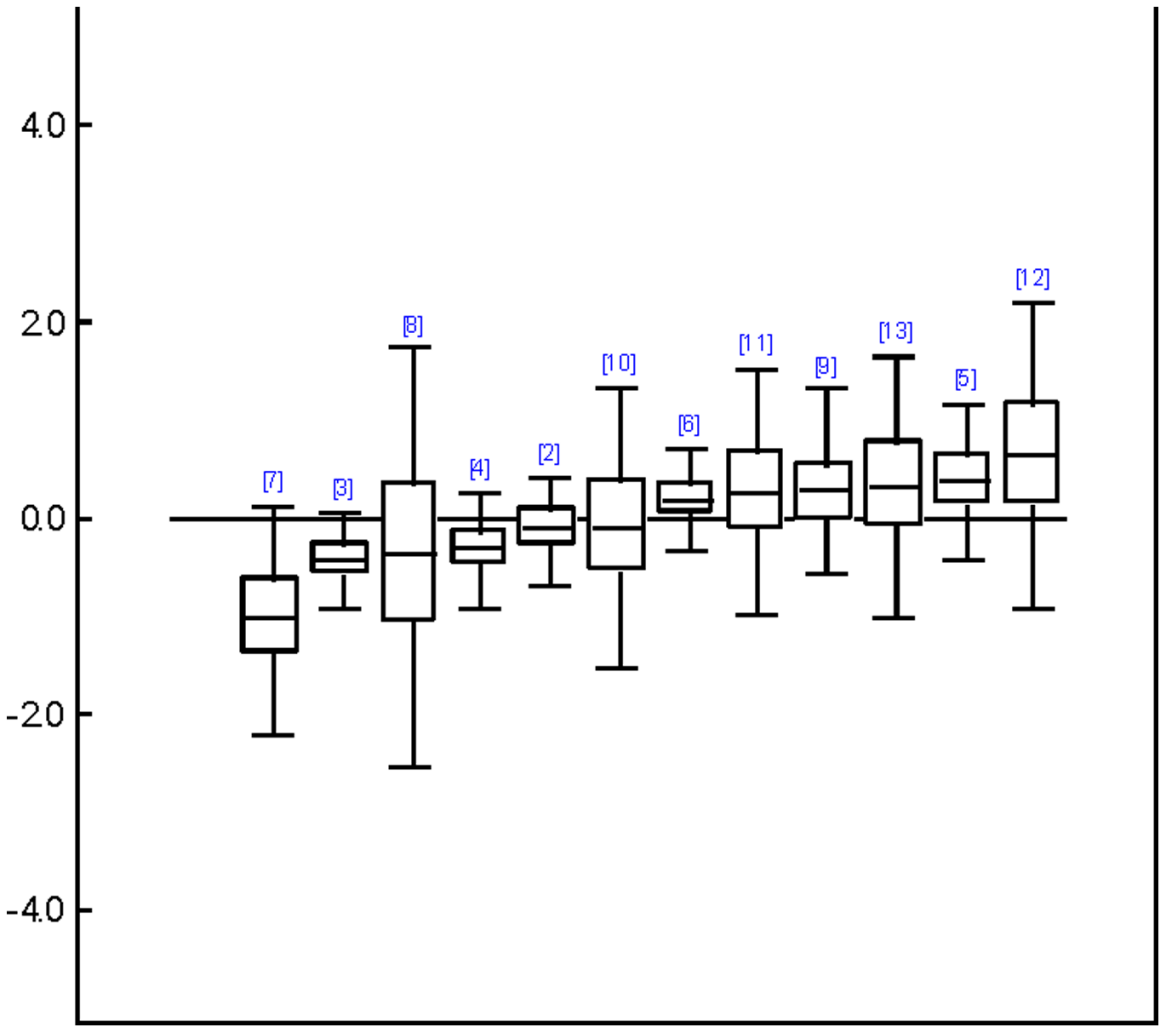
Relative efficacy of different treatments compared to cognitive behavior therapy referring to clinical significance. Relative differences are presented on a log odds ratio scale with the solid line representing cognitive behavior therapy as reference ( = 1). 2 =  behavior activation therapy; 3 =  supportive therapies; 4 =  psychodynamic therapy; 5 =  problem solving therapy; 6 =  interpersonal therapy; 7 =  social skills training; 8 =  acceptance and commitment therapy; 9 =  cognitive behavioral analysis system of psychotherapy; 10 =  coping-oriented couples therapy; 11 =  process-experiential therapy; 12 =  self-system therapy; 13 =  emotion focused therapy.

In Type II analyses, those types of therapies which had been grouped into “other therapies” before, could be analyzed separately as well, resulting in 19, 15 and 13 different types of treatments, respectively which could be compared concerning patient self-ratings, clinician ratings and their clinical significance (see Appendix S3 for WinBUGS code used). Results of relative treatment differences are shown in Figures 3, 4, and 5, using CBT as a common comparison treatment. Even though confidence intervals were quite large and results did not reach nominal significance with regard to patient self-ratings and clinical significance, interesting trends could be observed. Overall, SUP and DYN tended to be somewhat less efficacious than CBT whereas BA and IPT performed approximately equal to CBT. Across all outcomes, cognitive behavioral analysis system of psychotherapy (CBASP), problem-solving therapy (PST), and especially self-system therapy came out on top. Note, however, that only two direct comparisons were available for CBASP and only one for self-system therapy. The status of social skills training (SST) was ambiguous because it belonged to the probably best treatments concerning patient self-ratings and clinician ratings (*g* = 0.47 and *g* = 0.91 vs. CBT; the difference was even significant concerning clinician ratings as the confidence interval did not contain the CBT-baseline), whereas it ended up as the worst treatment in terms of clinical significance. Overall, rankings of treatments concerning patient self-ratings and clinician ratings were highly correlated (Spearman *r* = .67, *p* = .007). However, these rankings did not correlate with rankings concerning clinical significance (*r* = .07 and *r* = –.20, *p* = .814 and.540). Treatments whose rankings were most discrepant across outcomes (i.e., exceeded half the number of compared treatments) were CBASP, SST, and ACT. For these treatments only two to four comparisons were available each.

Addressing the comparisons of CBT versus SUP, BA, DYN and IPT Table 7 displays a comparison between the results of Type I and Type II meta-analyses. Overall, results pointed to the same direction; for supportive therapies, effect sizes of Type II meta-analysis were somewhat larger. Note, however, that none of these Type II meta-analysis results were statistically significant.

**Table 7:** Comparison of the Results of Type I and Type II Meta-Analyses.

	Patient self-ratings		Clinician ratings		Clinical Significance		
Cognitive behavior therapy vs.	Type I	Type II	Type I	Type II	Type I		Type II
Supportive therapies	–0.05 [–0.21, 0.12]	–0.18 [–0.39, 0.03]	–0.08 [–0.46, 0.30]	–0.20 [–0.60, 0.18]	1.49 [0.81, 2.73]	1.53 [0.95, 2.49]	
Behavior activation therapy	–0.06 [–0.39, 0.27]	0.08 [–0.17, 0.30]	–0.25 [–0.66, 0.16]	–0.10 [–0.46, 0.23]	1.03 [0.45, 2.35]	1.12 [0.66, 2.01]	
Psychodynamic therapy	–0.16 [–0.36, 0.04]	–0.14 [–0.40, 0.09]	–0.08 [–0.40, 0.25]	0.04 [–0.38, 0.46]	1.04 [0.48, 2.24]	1.38 [0.79, 2.49]	
Interpersonal therapy	0.08 [–0.16, 0.33]	–0.03 [–0.28, 0.23]	0.00 [–0.30, 0.30]	0.00 [–0.36, 0.33]	0.85 [0.49, 1.47]	0.84 [0.49, 1.39]	

*Note.* 95% confidence intervals (Type I) and 95% Bayesian confidence interval/credible interval (Type II) are provided in brackets. Figures correspond to *g* for patient self-ratings and clinician ratings, and to *OR* for clinical significance. Negative effect sizes (*g*) indicate that the focus treatment was more efficacious than the treatment compared. *OR*>1 indicates that the odds of remission in the focus treatment were higher than in the treatment compared.

## Discussion

The purpose of this study was to investigate whether all bona fide psychotherapies are equally efficacious in the treatment of depression. Using two different meta-analytical approaches and an updated study sample we were able to clarify some conflicting previous findings and to accentuate some new findings. While differences in efficacy tended to be small overall, we found some indication that patient characteristics and therapy format may play important roles for some treatments.

Regarding Type I meta-analyses, supportive therapies were somewhat less efficacious than other treatments at posttest. This corroborates previous findings [5]. However, while this has been previously explained by the use of supportive therapies as a mere control condition, we only included treatments in our study that were therapeutically intended and thus bona fide. The overall effect appeared to be rather small (*g* = 0.36 in clinician ratings for supportive therapies not referring to Rogers; *g* = 0.28 in patient self-ratings and *OR* = 0.61 concerning the clinical significance of changes for therapies referring to Rogers) and was possibly also affected by publication bias. However, in samples of “purely” depressed patients, supportive therapies remained consistently less efficacious than other therapies with regard to clinical significance. Cast into numbers-needed-to-treat (NNT; using [37]), the NNT was 9 (95% confidence interval = [6], [36]) in these studies for one extra patient to achieve a clinically significant change when treated with another treatment compared to supportive therapy. Consequently, our results suggest that supportive therapies might not be as effective as other therapies in the treatment of depression even when they have to be considered as bona fide. Proportion of real heterogeneity of effects sizes (*I* ^2^) was low in these analyses, indicating that our findings were not due to outlying studies but systematic. On the other side, confidence intervals for *I* ^2^ and thus uncertainty regarding this measure were quite large. Moreover, in specific comparisons with CBT, supportive therapies did not fare any worse. Hence, the status of supportive therapies within the treatment of depressive disorders needs to be further clarified in future studies. Similarly, obtained evidence on the lower efficacy of psychodynamic therapies in the treatment of “purely” depressed patients needs to be handled with caution. Reporting bias was found to impact the efficacy of psychodynamic therapies (discussed below). Therefore, more high quality studies are needed to clarify the status of psychodynamic therapies as well.

Concerning time of follow-up, results were again consistent with [5]: Efficacy of all treatments seemed comparable. However, there was a great range of time to follow-up, reaching from one to 24 months. Because this investigation did not concentrate on differences between treatments at time of follow-up, moderator analyses were not conducted with regard to time of follow-up. Hence, these results should be interpreted with caution and should be analyzed in more detail in the future especially having in mind that depression is a disorder which occurs episodically.

Chances of completing treatment were lower in cognitive therapy according to Beck (*OR* = 0.77; NNT = 23 [12, 212] for other therapies to retain one extra patient compared to cognitive therapy according to Beck) but higher in all other cognitive behavior therapies (*OR* = 1.95; NNT = 12 [8, 127] for cognitive behavior therapy to retain one extra patient compared to other treatments) and in interpersonal therapy (*OR* = 1.65; NNT = 13 [9], [45] for interpersonal therapy to retain one extra patient compared to other treatments), contrasting previous findings that did not differentiate between subtypes of cognitive behavior therapies with regard to drop-out [5]. Differences in drop-out might reflect differences in acceptance of therapies. However, it is not clear why the two subtypes of cognitive behavior therapy differed with regard to drop-out. The number of comparisons on cognitive therapy according to Beck was large (*k* = 36), providing enough statistical power to deem results reliable. However, NNT was also large, indicating that the effect is likely negligible in practice. Chances of completing treatment were also lower in psychodynamic therapy (*OR* = 0.70; NNT = 18 for other therapies to retain one extra patient compared to psychodynamic therapy), but did not reach significance there because of comparably less available studies (*k* = 12) and thus low power [5]. Moreover, NNT was large again, suggesting that this effect was also practically negligible. Definition of drop-out differed considerably between studies which might have contributed to our findings. Yet, attrition needs to be considered seriously with regard to the clinical applicability of treatments. Our findings suggest that treatments of depression differ in terms of their applicability and that patients accept especially cognitive behavior therapy and interpersonal therapy better than other treatments.

Moderator analyses revealed that several variables were associated with effect size. First, psychodynamic therapies performed significantly worse when there was a high risk for reporting bias. Even though there was no indication that psychodynamic therapy was less efficacious overall, this finding suggests that study quality may bias primary studies with regard to specific treatments systematically and needs to be carefully considered in primary research.

Second, behavior activation therapy was more efficacious than other treatments in samples of older patients. This is consistent with findings of other studies [38], [39]. Patient characteristics, like age, thus need to be considered more closely with regard to recommendations of treatment: Behavior activation therapy may be especially effective for older patients. However, at the same time, behavior activation therapy appeared to be less efficacious than other treatments when it was delivered in a group or couples format. Adapting interventions to the patient in an individual treatment format may be especially beneficial as it comes to the development of specific behavior patterns which might be strongly influenced by personal preferences and habits. Although behavior activation therapy has often been considered to be a treatment which is especially feasible in the group format [40], this may need to be reconsidered. Mean age of patients and format of therapy were confounded and only few studies with a group or couples format were available. Hence, more studies are needed to separate the effects of treatment format and participant age with regard to behavior activation therapy more clearly.

Third, cognitive behavior therapy was more efficacious and behavior activation therapy was less efficacious than other treatments in samples with higher proportions of female patients. Even though proportion of female patients was confounded with publication year and whether therapists conducted both types of treatments in studies on cognitive behavior therapy, these factors did not influence the observed association. Therefore, our results point at reliable gender differences in treatment efficacy. A recent study [41] showed that female patients may benefit more from cognitive behavior therapy than male patients in the treatment of compulsive-obsessive disorder. Another study [42] reported that interpersonal therapy of depression was more efficacious in men – a finding that could not, however, be reliably replicated in our study. The causes of gender differences in efficacy are currently unclear. However, gender differences in treatment efficacy are clearly understudied. More research is needed on this issue.

Forth, cognitive behavior therapy also proved to be more efficacious than other treatments in samples with higher rates of comorbid anxiety disorders. Although this finding was only based on a few studies, these studies likely had overall higher standards as all of them incorporated checks on the adherence to treatment manuals. Moreover, this finding is consistent with previous research, which has shown that depressive patients with a comorbid anxiety disorder improved faster than patients who suffered only from depression [43]. Also, cognitive behavior therapy was considered as an effective treatment regarding anxiety disorders in some meta-analyses [4], [44]. More research is needed on the effect of comorbidity on treatment outcome. Yet, some treatments seem to be more efficacious with regard to different types of comorbid disorders, which may affect recommendations of treatment.

An interesting finding of this study pertained to cognitive behavior therapy being superior to all other treatments in studies where therapy sessions lasted 90 minutes or longer. These results are consistent with a meta-analysis on cognitive group therapy of depression [45]. Conversely, behavior activation therapy was more efficacious when therapy sessions lasted less than 90 minutes. Even though length of therapy session was confounded with format of therapy such that therapies with longer sessions were more often conducted as group therapies, results remained similar when comparisons were restricted to therapies conducted in an individual format. Also, length of therapy sessions was confounded with type of analysis for cognitive behavior therapy. However, confounding did not moderate effects of session length. Still, these results should be treated with caution; it is still not clear how format and length of therapy session contribute to the efficacy of a certain therapy. To our knowledge currently no other studies exist which have investigated the optimal length of therapy sessions. In clinical applications, length of therapy session is mostly determined by institutional conditions and constraints. However, according to the concept of sudden gains in cognitive behavior therapy [46], it is the changes in the client that occur during therapy session that are responsible for general clinical change. Hence, length of therapy session may be a crucial factor that needs to be considered and investigated more closely, especially with regard to more complex and demanding treatments such as cognitive behavior therapy. Judging from our results, shorter therapy sessions may confer a specific advantage to behavior activation therapy. However, this may render comparisons with other treatments in an individual format unfair, as these are most often administered in therapy sessions of less than 90 minutes. Length of therapy session may thus need to be considered more closely in meta-analytical investigations regarding the efficacy of different treatments but also in primary research.

Even though most results within mixed treatment comparisons did not reach level of significance supportive therapies tended to be less efficacious than the other treatments. Yet, it was noticeable that none of the “classical” treatments emerged as the probable most efficacious in these analyses. In contrast, mostly recently developed treatments were among the probable best treatments: Self-system therapy and cognitive behavioral analysis system of psychotherapy. Both treatments were specifically developed for the treatment of depressive disorders. However, more studies are needed with regard to these treatments; clearly, it is not possible to draw final conclusions about the efficacy of these treatments considering the small study sample size. It is also to be seen whether results will be subject to a decline effect: Positive effects of a new type of treatment in earlier studies may disappear in later studies [47]. Problem solving therapy emerged among the most efficacious treatments as well which is consistent with findings in other meta-analyses that were not confined to bona fide treatments [48], [49]. Social skills training emerged among the probable best treatments concerning patient self-ratings and clinician ratings, whereas as the worst treatment concerning clinical significance. Impaired social skills are often associated with depressive disorders [50]. However, the ambiguity of the results in this study and evidence of only four studies suggests that the efficacy of social skills training in the treatment of depression needs further investigation. Notably, clinical significance was determined with rather strict criteria in the only two studies on social skills training where tests on it were provided: Both BDI scores as well as HRSD scores had to be below 9 and 7, respectively, or below 10. Across all studies, 21 different criteria were used to categorize patients as remitted, including the use of thresholds concerning patient self-ratings, clinician ratings or both as well as diagnostic criteria (i.e., absence of a diagnosis of depressive disorders). In general, results with regard to clinical significance did not agree with results on patient self-ratings and clinician ratings that appeared to match acceptably with one another. Similar to drop-out (see above), encountered varying definitions of clinical significance thus likely limit the informative value of respective results. Future meta-analyses may need to strictly limit their analyses on certain, more concordant criteria.

This study has several important limitations. First, the number of studies was not sufficient to conduct separate meta-analyses for each type of treatment and to systematically investigate all pairs or triplets of moderators in meta-regression analyses. Therefore, some potential interactions between several moderator variables could not be examined. Moreover, moderators may have also been confounded with unmeasured variables. Also, some direct comparisons were infeasible. For example, there was no single study which compared psychodynamic therapy with interpersonal therapy. Second, study quality was assessed in detail, but it could only be implemented in moderator analyses by recoding “unclear risk of bias” to “high risk of bias” due to the small sample sizes in the three categories of the ratings in the risk of bias assessment tool and insufficient information in primary studies. Furthermore, study quality was coded by only one author. Third, although differences in allegiance were not related to effect size, it is not clear whether our operationalization reflected reliably true differences in allegiance [22]. Coding of this variable was also only executed once. Fourth, classification of treatments was conducted according to their naming in the respective study. Future research might want to employ an expert classification procedure as it has been done by [51] to account for the fact that implementation of various treatments may differ from study to study despite identical designation. Fifth, results differed across types of outcome measures. It may be unclear or ambiguous which type of outcome measure may be the most adequate to represent relevant differences between treatments. Our study suggests that definitions underlying clinical significance and possibly also drop-out may be too heterogeneous across studies to allow meaningful meta-analytical investigations. Moreover, apart from drop-out, outcome measures that did not directly refer to disorder-specific symptom improvement were not considered in this study (i.e., non-targeted or global measures). Treatments might or might not differ with regard to such alternative measures [51]. Sixth, the potential impact of studies which were excluded because of insufficient information or of unpublished studies on our results is not clear. As mentioned above, tests for publication bias revealed that unpublished studies likely impacted some findings. According to Trinquart et al. [52] publication bias especially affects results of network meta-analyses as the overall ranking of treatments and not only the effect size of the focus treatment might be biased. Because presence of publication bias was revealed by funnel plots in some cases, the results of the mixed treatment comparisons must be treated with some caution. Last but not least, both conventional meta-analysis as well as mixed treatment comparisons face several methodological limitations, see [31] for a discussion. Mixed treatment comparisons may yield differing results even when applied to similar datasets [18], [53]. Especially when quality of the primary studies is considered to be problematic, meta-analytical findings need to be treated with caution. In our study, patient characteristics were confounded with treatment effects, questioning the validity of summarizing a research field by single numbers [31]. Direct and indirect comparisons may have thus been inconsistent to some degree with regard to some treatments, possibly biasing the overall result of the analysis.

Despite the limitations of our study, our results suggest that the dodo bird verdict seems to be the right answer for the wrong question. Even though it seemed mostly corroborated at the aggregate level, there appear to exist a number of differential effects in efficacy between bona fide treatments of depression at a finer level. Future research should address patient characteristics, like gender, age or comorbid mental disorders, more explicitly and determine which treatments work best with which group of patients [24]. Furthermore, an empirical and theoretical framework is needed that may explain differences in efficacy. Research also needs to focus on length of therapy session. Despite being mostly determined by institutional restrictions, length of therapy session may be a treatment characteristic with genuine effects. Mixed treatment comparisons revealed that recently developed treatments showed promise with regard to their efficacy in our study. This indicates that the development of potent treatments for depression is not yet at its end. However, both types of meta-analyses yielded slightly different results. This must be taken into account in judging about the relative efficacy of psychotherapies: Conclusions which are based on only one approach may not comprise the most sufficient aggregation of the available evidence and hence may need to be interpreted with caution.

## Supporting Information

Table S1 **Descriptive information on included studies.**
(DOCX)

Table S2 **Results of individual studies.**
(DOCX)

Appendix S1 **Studies included in the meta-analyses.**
(DOCX)

Appendix S2 **Studies that were excluded in the last step.**
(DOCX)

Appendix S3 **WinBUGS code used for mixed treatment comparisons.**
(DOCX)
