# Supplementary material for: Comparing Bona Fide Psychotherapies of Depression in Adults with Two Meta-Analytical Approaches
Source: PLoS One. 2013 Jun 28;8(6):e68135. doi: 10.1371/journal.pone.0068135 (PMC3695954; doi:10.1371/journal.pone.0068135)
Supplement: Table S1 — Descriptive information on included studies. (DOCX) [file pone.0068135.s001.docx]

Table S1

*Descriptive Information on Included Studies*

|  |  |  |  | Included in | Outcome measure | | Time to | Risk of bias |
| --- | --- | --- | --- | --- | --- | --- | --- | --- |
| Study | *N* | Mean age of participants | Compared Treatments | meta-analyses | Patient self-rating | Clinician rating | follow-up (in months) |  |
| Alladin & Alibhai (2007) | 98 | 35.62 | CBT (according to Beck) vs. Cognitive hypnotherapy | --- | BDI-II | --- | 12 | ?/?/L/H/H/L |
| Arean et al. (2010) | 221 | 73.00 | Problem solving therapy vs. SUP | 1, 3 | --- | HRSD-17 | --- | L/L/L/L/?/L |
| Barkham et al. (1999) | 116 | 44.90 | CBT (according to Beck) vs. DYN | 3 | BDI | --- | 12 | ?/?/L/H/?/L |
| Beutler et al. (1991) | 55 | 46.76 | CBT (according to Beck) vs. Process-experiential therapy | 1, 2, 3 | BDI | HRSD-17 | 1.5 | ?/L/?/L/L/L |
| Beutler et al. (2003) | 40 | 33.06 | CBT (according to Beck) vs. Cognitive-narrative therapy vs. Prescriptive therapy | 1 | BDI | HRSD-17 | 6 | ?/L/?/L/L/L |
| Bodenmann et al. (2008) | 60 | 45.30 | CBT (according to Beck) vs. Coping-oriented couples therapy vs. IPT | --- | BDI | HRSD-17 | 18 | ?/L/L/?/L/L |
| Bright et al. (1999) | 55 | 45.80 | CBT (according to Beck) vs. SUP | 1 | BDI | HRSD (revised) | --- | ?/L/L/?/H/? |
| Comas-Díaz (1981) | 32 | 38.00 | CBT (according to Beck) vs. BA | 1 | --- | HRSD-17 | 1 | ?/H/?/L/H/H |
| Cooper et al. (2003) | 141 | 28.10 | CBT vs. DYN vs. SUP | --- | EPDS | --- | 60 | L/?/L/H/L/L |
| David et al. (2008) | 113 | 37.00 | CBT (according to Beck) vs. CBT | --- | BDI-II | HRSD-17 | 6 | ?/L/L/L/L/L |
| Dimidjian et al. (2006) | 88 | 39.90 | CBT (according to Beck) vs. BA | 1 | BDI-II | HRSD (modified by the authors) | --- | L/L/L/L/L/L |
| Elkin et al. (1989) | 120 | 35.00 | CBT (according to Beck) vs. IPT | 1, 2, 3 | BDI | HRSD-17 | 12 | L/L/L/L/L/L |
| Evans & Connis (1995) | 52 | 54.00 | CBT (according to Beck) vs. SUP | 1 | CES-D | --- | 6 | ?/?/L/H/?/? |
| Forman et al. (2007) | 99 | 27.90 | Acceptance and commitment therapy vs. CBT (according to Beck) | --- | BDI-II | --- | --- | ?/L/L/?/H/L |
| Gallagher & Thompson (1982) | 38 | 67.80 | BA vs. CBT (according to Beck) vs. Brief relational insight therapy | 1, 2 | BDI | HRSD-17 | 12 | ?/L/L/H/L/L |
| Gallagher-Thompson & Steffen (1994) | 66 | 62.00 | CBT (according to Beck) vs. DYN | 1, 2, 3 | BDI | HRSD-17 | 3 | ?/L/?/H/?/? |
| Goldman et al. (2006) | 42 | 39.50 | Emotion focused therapy vs. SUP (referring to Rogers) | --- | BDI | --- | --- | ?/L/L/?/L/L |
| Greenberg & Watson (1998) | 34 | 39.64 | Process-experiential therapy vs. SUP (referring to Rogers) | --- | BDI | --- | 6 | ?/L/L/?/L/L |
| Hersen et al. (1984) | 64 | 30.40 | DYN vs. Social skills training | 1 | BDI | HRSD-17 | --- | ?/L/L/H/H/? |
| Hogg & Deffenbacher (1988) | 27 | 23.14 | CBT (according to Beck) vs. Interpersonal process therapy | 1, 2 | BDI | --- | 1.25 | ?/L/L/?/?/L |
| Hopko et al. (2011) | 80 | 55.40 | BA vs. Problem solving therapy | --- | BDI-II | HRSD-17 | 12 | L/L/?/L/?/L |
| Jacobson et al. (1991) | 50 | 38.50 | BA vs. CBT (according to Beck) | 1 | BDI | HRSD-17 | --- | ?/L/?/L/L/L |
| Kelly et al. (1993) | 77 | 34.00 | CBT vs. SUP | 1 | CES-D | --- | 3 | ?/H/L/H/?/L |
| King et al. (2000) | 260 | 36.80 | CBT vs. SUP (referring to Rogers) | 1 | BDI | --- | 12 | L/?/L/L/L/L |
| Kiosses et al. (2010) | 30 | 79.40 | Problem solving therapy vs. SUP |  | --- | HRSD-24 | --- | ?/H/L/?/L/L |
| Kocsis et al. (2009) | 395 | 46.00 | Cognitive behavioral analysis system of psychotherapy vs. SUP (referring to Rogers) | --- | --- | HRSD-24 | --- | L/L/L/?/L/L |
| Kornblith et al. (1983) | 22 | 37.90 | BA vs. DYN | --- | BDI | HRSD-17 | 3 | ?/?/L/?/L/L |
| Koszycki et al. (2012) | 31 | 35.50 | IPT vs. SUP (referring to Rogers) | --- | BDI-II | --- | 6 | ?/L/?/L/?/L |
| Luty et al. (2007) | 177 | 35.20 | CBT (according to Beck) vs. IPT | --- | BDI-II | HRSD-17 | 6 | L/L/L/L/L/L |
| Maina et al. (2005) | 20 | 40.00 | DYN vs. SUP | 1 | --- | HRSD-17 | 6 | ?/?/L/L/H/H |
| Manicavasgar et al. (2011) | 61 | 45.80 | CBT (according to Beck) vs. Mindfulness based cognitive therapy | --- | BDI-II | --- | 12 | L/L/L/?/L/L |
| Markowitz et al. (1998) | 75 | 36.90 | CBT (according to Beck) vs. IPT vs. SUP (referring to Rogers) | 1 | BDI | HRSD-17 | --- | L/L/L/?/?/L |
| Markowitz et al. (2005) | 49 | 42.30 | IPT vs. SUP (referring to Rogers) | 1 | BDI | HRSD-24 | --- | L/L/L/L/?/L |
| Marshall et al. ( 2008) | 72 | --- | CBT vs. IPT | --- | --- | HRSD-17 | --- | ?/?/?/?/H/L |
| McLean & Hakstian (1979) | 77 | 39.20 | BA vs. DYN | 1, 2, 3 | BDI | --- | 3 | ?/?/L/H/?/H |
| McNamara & Horan (1986) | 37 | 23.00 | BA vs. CBT (according to Beck) vs. SUP (referring to Rogers) | 1 | BDI | --- | 2 | ?/?/L/H/H/L |
| Milgrom et al. (2005) | 159 | 29.70 | BA vs. SUP vs. SUP | 1 | BDI | --- | 12 | H/L/L/?/H/L |
| Miller et al. (1989) | 29 | 35.20 | CBT (according to Beck) vs. Social skills training | --- | BDI | HRSD-25 | --- | ?/?/?/?/?/L |
| Mohr et al. (2001) | 42 | 43.90 | CBT vs. SUP | 1 | BDI | HRSD-17 | 6 | H/L/H/H/?/L |
| O'Leary & Beach (1990) | 30 | 39.30 | CBT (according to Beck) vs. Problem solving therapy | --- | BDI | --- | --- | ?/?/L/H/H/L |
| Power & Freeman (2012) | 100 | 36.10 | CBT (according to Beck) vs. IPT | --- | BDI-II | HRSD-24 | --- | ?/L/L/L/H/L |
| Rude (1986) | 32 | 40.00 | CBT vs. Social skills training | 1 | BDI | --- | --- | ?/?/L/H/H/L |
| Sanchez et al. (1980) | 32 | --- | DYN vs. Social skills training | 1 | BDI | --- | 1 | ?/?/L/H/H/L |
| Schramm et al. (2011) | 29 | 40.20 | Cognitive behavioral analysis system of psychotherapy vs. IPT | --- | BDI | HRSD-24 | 12 | L/L/L/L/L/L |
| Shapiro et al. (1994) | 75 | 40.50 | CBT (according to Beck) vs. DYN | 2, 3 | BDI | --- | 12 | ?/?/L/H/?/L |
| Shaw (1977) | 16 | 19.90 | BA vs. CBT (according to Beck) | 1 | BDI | HRSD-17 | 1 | ?/H/L/L/?/L |
| Steuer et al. (1984) | 33 | 66.00 | CBT (according to Beck) vs. DYN | 2 | BDI | HRSD-21 | --- | ?/L/?/?/?/L |
| Strauman et al. (2006) | 45 | 39.40 | CBT (according to Beck) vs. Self-system therapy | 1 | BDI | HRSD-17 | --- | L/L/L/L/L/L |
| Teri et al. (1997) | 42 | 76.40 | BA vs. Problem solving therapy | 1 | BDI | HRSD-17 | 6 | ?/?/L/?/?/L |
| Thompson et al. (1987) | 109 | 66.90 | BA vs. CBT (according to Beck) vs. DYN | 1, 2, 3 | BDI | HRSD-17 | 24 | ?/L/?/?/?/L |
| Watson et al. (2003) | 93 | 41.52 | CBT (according to Beck) vs. Process-experiential therapy | --- | BDI | --- | --- | L/L/L/?/L/L |
| Wilson et al. (1983) | 16 | 39.50 | BA vs. CBT (according to Beck) | 1 | BDI | HRSD-17 | 5 | ?/L/H/H/?/L |
| Zettle & Rains (1989) | 25 | 41.30 | Acceptance and commitment therapy vs. CBT (according to Beck) | 1 | BDI | HRSD-17 | 2 | ?/L/L/?/?/L |

*Note. N* = Number of participants. BA = behavior activation therapy, CBT = cognitive behavior therapy, DYN = psychodynamic therapy, IPT = interpersonal therapy, SUP = supportive therapies. 1 = Cuijpers, van Straten, Andersson, and van Oppen (2008); 2 = Wampold, Minami, Baskin, and Tierney (2002), 3 = Tolin (2010). BDI = Beck depression inventory, HRSD = Hamilton rating scale of depression, CES-D = Center for epidemiological studies depression scale, EPDS = Edinburgh postnatal depression scale. Study quality was assessed by means of the *risk of bias assessment tool* (Higgins, Altman, & Sterne, 2008); figures correspond to H = *high*, L = *low* and ? = *unclear* risk concerning bias with regard to selection, performance, detection, attrition, reporting, and other bias (in this order). Time to follow-up pertains to the longest time-span reported in the respective study. If time is not denoted, there was no follow-up assessment, time to follow-up was either not reported or results were not reported or rather not presented for each treatment group independently.
