# Supplementary material for: Comparing Bona Fide Psychotherapies of Depression in Adults with Two Meta-Analytical Approaches
Source: PLoS One. 2013 Jun 28;8(6):e68135. doi: 10.1371/journal.pone.0068135 (PMC3695954; doi:10.1371/journal.pone.0068135)
Supplement: Table S2 — Results of individual studies. (DOCX) [file pone.0068135.s002.docx]

Table S2

*Results of Individual Studies*

|  | | |  | | Outcome measure | | | | |
| --- | --- | --- | --- | --- | --- | --- | --- | --- | --- |
| Study | *n/n* | Compared Treatments | Patient self-rating | Clinician rating | | Combined | Clinical significance | Definition of remission | Drop-Out |
| Alladin & Alibhai (2007) | 49/49 | CBT (according to Beck) vs. Cognitive hypnotherapy | 0.48 [0.04, 0.91] | --- | | --- | --- | --- | 1.00 [0.32, 3.10] |
| Arean et al. (2010) | 110/111 | Problem solving therapy vs. SUP | --- | -0.38 [-0.65, -0.12] | | --- | 2.17 [1.18, 3.98] | HRSD < 10 | 0.65 [0.31, 1.36] |
| Barkham et al. (1999) | 62/54 | CBT (according to Beck) vs. DYN | -0.13 [-0.50, 0.24] | --- | | --- | --- | --- | --- |
| Beutler et al. (1991) | 27/28 | CBT (according to Beck) vs. Process-experiential therapy | -0.06 [-0.67, 0.53] | -0.19 [-0.79, 0.41] | | -0.13 [-0.63, 0.37] | --- | --- | 1.07 [0.37, 3.09] |
| Beutler et al. (2003) | 15/13 | CBT (according to Beck) vs. Cognitive-narrative therapy | -0.50 [-1.30, 0.30] | -0.41 [-1.54, 0.72] | | -0.46 [-1.27, 0.36] | --- | --- | 0.54 [0.04, 6.77] |
|  | 15/12 | CBT (according to Beck) vs. Prescriptive therapy | -0.57 [-1.39, 0.25] | -0.32 [-1.34, 0.70] | | -0.44 [-1.22, 0.33] | --- | --- | 0.59 [0.05, 7.43] |
|  | 13/12 | Cognitive-narrative therapy vs. Prescriptive therapy | -0.11 [-0.93, 0.71] | -0.01 [-1.16, 1.13] | | -0.06 [-0.89, 0.76] | --- | --- | 1.09 [0.06, 19.63] |
| Bodenmann et al. (2008) | 20/20 | CBT (according to Beck) vs. Coping-oriented couples therapy | -0.04 [-0.67, 0.59] | 0.07 [-0.56, 0.69] | | 0.01 [-0.51, 0.54] | 0.92 [0.25, 3.42] | diagnosis of depression not present anymore | 2.22 [0.07, 70.28] |
|  | 20/20 | CBT (according to Beck) vs. IPT | -0.10 [-0.73, 0.54] | 0.67 [-0.57, 0.70] | | -0.02 [-0.55, 0.52] | 0.61 [0.16, 2.28] | diagnosis of depression not present anymore | 4.44 [0.19, 105.15] |
|  | 20/20 | Coping-oriented couples therapy vs. IPT | -0.06 [-0.70, 0.59] | -0.01 [-0.66, 0.63] | | -0.03 [-0.57, 0.51] | 0.66 [0.17, 2.49] | diagnosis of depression not present anymore | 2.11 [0.18, 25.35] |
| Bright et al. (1999) | 27/28 | CBT (according to Beck) vs. SUP | -0.29 [-0.92, 0.34] | -0.05 [-0.67, 0.57] | | -0.17 [-0.69, 0.35] | 3.41 [1.10, 10.56] | BDI ≤ 10 | 0.55[0.16, 1.82] |
| Comas-Díaz (1981) | 16/16 | CBT (according to Beck) vs. BA | --- | -1.12 [-2.19, -0.04] | | --- | --- | --- | 1.00 [0.02, 53.56] |
| Cooper et al. (2003) | 43/50 | CBT vs. DYN | 0.07 [-0.35, 0.49] | --- | | --- | 0.54 [0.22, 1.32] | diagnosis of depression not present anymore | 5.13 [1.06, 24.87] |
|  | 43/48 | CBT vs. SUP | -0.13 [-0.55, 0.29] | --- | | --- | 1.13 [0.49, 2.60] | diagnosis of depression not present anymore | 2.93 [0.56, 15.36] |
|  | 50/48 | DYN vs. SUP | -0.19 [-0.60, 0.22] | --- | | --- | 2.08 [0.88, 4.92] | diagnosis of depression not present anymore | 0.57 [0.19, 1.72] |
| David et al. (2008) | 56/57 | CBT (according to Beck) vs. CBT | 0.00 [-0.37, 0.37] | -0.03 [-0.40, 0.34] | | -0.01 [-0.32, 0.30] | 1.28 [0.61, 2.68] | diagnosis of depression not present anymore + HRSD < 7 | 0.80 [0.23, 2.79] |
| Dimidjian et al. (2006) | 45/43 | CBT (according to Beck) vs. BA | 0.29 [-0.20, 0.79] | 0.15 [-0.35, 0.64] | | 0.22 [-0.20, 0.63] | 0.92 [0.40, 2.12] | BDI ≤ 10 | 1.26 [0.39, 4.12] |
| Elkin et al. (1989) | 59/61 | CBT (according to Beck) vs. IPT | 0.13 [-0.23, 0.49] | 0.11 [-0.25, 0.47] | | 0.12 [-0.18, 0.42] | 0.77 [0.37, 1.57] | BDI ≤ 9 | 0.63 [0.28, 1.41] |
| Evans & Connis (1995) | 29/23 | CBT (according to Beck) vs. SUP | 0.21 [-0.37, 0.78] | --- | | --- | --- | --- | 1.29 [0.17, 9.90] |
| Forman et al. (2007) | 55/44 | Acceptance and commitment therapy vs. CBT (according to Beck) | 0.01 [-0.39, 0.41] | --- | | --- | --- | --- | 1.44 [0.64, 3.25] |
| Gallagher & Thompson (1982) | 15/11 | BA vs. CBT (according to Beck) | 0.30 [-0.59, 1.18] | 0.04 [-0.83, 0.92] | | 0.17 [-0.57, 0.91] | --- | --- | 0.20 [0.02, 2.03] |
|  | 15/12 | BA vs. Brief relational insight therapy | -0.14 [-1.02, 0.73] | -0.43 [-1.32, 0.46] | | -0.29 [-1.03, 0.45] | --- | --- | 0.40 [0.06, 2.57] |
|  | 11/12 | CBT (according to Beck) vs. Brief relational insight therapy | -0.51 [-1.40, 0.38] | -0.69 [-1.60, 0.22] | | -0.60 [-1.36, 0.15] | --- | --- | 2.00 [0.16, 25.76] |
| Gallagher-Thompson & Steffen (1994) | 36/30 | CBT (according to Beck) vs. DYN | -0.39 [-0.95, 0.17] | -0.18 [-0.74, 0.37] | | -0.29 [-0.75, 0.18] | 2.11 [0.62, 7.14] | diagnosis of depression not present anymore | 2.66 [0.78, 9.05] |
| Goldman et al. (2006) | 21/21 | Emotion focused therapy vs. SUP (referring to Rogers) | -0.64 [-1.29, 0.02] | --- | | --- | 1.73 [0.40, 7.51] | diagnosis of depression not present anymore + BDI ≤ 8 | 1.00 [0.13, 7.85] |
| Greenberg & Watson (1998) | 17/17 | Process-experiential therapy vs. SUP (referring to Rogers) | -0.13 [-0.80, 0.54] | --- | | --- | 2.28 [0.52, 9.99] | BDI ≤ 10 | 1.00 [0.12, 8.06] |
| Hersen et al. (1984) | 31/33 | DYN vs. Social skills training | 0.50 [-0.08, 1.08] | 0.31 [-0.27, 0.89] | | 0.40 [-0.08, 0.89] | 0.51 [0.18, 1.40] | BDI and HRSD ≤ 10 | 0.78 [0.26, 2.38] |
| Hogg & Deffenbacher (1988) | 13/14 | CBT (according to Beck) vs. Interpersonal process therapy | 0.06 [-0.70, 0.81] | --- | | --- | --- | --- | 0.93 [0.02, 50.30] |
| Hopko et al. (2011) | 42/38 | BA vs. Problem solving therapy | 0.14 [-0.30, 0.58] | -0.16 [-0.60, 0.28] | | -0.01 [-0.38, 0.36] | 0.68 [0.27, 1.70] | BDI ≤ 10 | 0.48 [0.15, 1.58] |
| Jacobson et al. (1991) | 27/23 | BA vs. CBT (according to Beck) | 0.51 [-0.13, 1.15] | 0.78 [-0.13, 1.44] | | 0.65 [-0.10, 1.19] | 0.54 [0.13, 2.34] | diagnosis of depression not present anymore + BDI < 10 | 0.36 [0.08, 1.55] |
| Kelly et al. (1993) | 39/38 | CBT vs. SUP | 0.27 [-0.38, 0.92] | --- | | --- | 0.58 [0.14, 2.34] | clinical significant change according to the reliable change index | 3.86 [1.50, 9.94] |
| King et al. (2000) | 134/126 | CBT vs. SUP (referring to Rogers) | 0.05 [-0.20, 0.29] | --- | | --- | --- | --- | 0.93 [0.51, 1.72] |
| Kiosses et al. (2010) | 15/15 | Problem solving therapy vs. SUP | --- | -0.75 [-1.49, -0.01] | | --- | --- | --- | 1.63 [0.23, 11.46] |
| Kocsis et al. (2009) | 200/195 | Cognitive behavioral analysis system of psychotherapy vs. SUP (referring to Rogers) | --- | 0.18 [-0.04, 0.39] | | --- | 1.38 [0.88, 2.16] | HRSD < 8 + reduction greater than 50% | 1.13 [0.63, 2.02] |
| Kornblith et al. (1983) | 16/6 | BA vs. DYN | 1.23 [0.06, 2.39] | 0.74 [-0.36, 1.84] | | 0.98 [0.04, 1.93] | 0.27 [0.01, 6.51] | diagnosis of depression not present anymore | 0.44 [0.04, 4.82] |
| Koszycki et al. (2012) | 15/16 | IPT vs. SUP (referring to Rogers) | -0.57 [-1.29, 0.15] | --- | | --- | 3.30 [0.75, 14.47] | HRSD ≤ 7 | 2.40 [0.47, 12.13] |
| Luty et al. (2007) | 86/91 | CBT (according to Beck) vs. IPT | -0.18 [-0.48, 0.11] | -0.22 [-0.51, 0.08] | | -0.20 [-0.45, 0.05] | 1.40 [0.77, 2.53] | HRSD ≤ 6 | 0.73 [0.27, 1.95] |
| Maina et al. (2005) | 10/10 | DYN vs. SUP | --- | -0.03 [-0.91, 0.85] | | --- | 1.50 [0.26, 8.82] | HRSD ≤ 7 + reduction greater than 50% | 1.00 [0.02, 55.52] |
| Manicavasgar et al. (2011) | 38/23 | CBT (according to Beck) vs. Mindfulness based cognitive therapy | -0.16 [-0.75, 0.44] | --- | | --- | --- | --- | 0.46 [0.13, 1.64] |
| Markowitz et al. (1998) | 27/24 | CBT (according to Beck) vs. IPT | 0.50 [-0.06, 1.06] | 0.59 [-0.03, 1.15] | | 0.55 [0.08, 1.02] | 0.39 [0.11, 1.36] | HRSD < 6 | 0.70 [0.22, 2.27] |
|  | 27/24 | CBT (according to Beck) vs. SUP (referring to Rogers) | -0.05 [-0.60, 0.50] | 0.20 [-0.35, 0.75] | | 0.08 [-0.38, 0.54] | 1.28 [0.33, 4.94] | HRSD ≤ 6 | 0.70 [0.22, 2.27] |
|  | 24/24 | IPT vs. SUP (referring to Rogers) | -0.54 [-1.11, 0.04] | -0.42 [-0.99, 0.15] | | -0.48 [-0.96, 0.00] | 3.30 [0.83, 13.18] | HRSD < 6 | 1.00 [0.29, 3.47] |
| Markowitz et al. (2005) | 23/26 | IPT vs. SUP (referring to Rogers) | 0.00 [-0.56, 0.56] | -0.16 [-0.72, 0.40] | | -0.08 [-0.55, 0.39] | 2.04 [0.43, 9.70] | HRSD ≤ 7 + reduction greater than 50% | 3.48 [0.92, 13.17] |
| Marshall et al. (2008) | 37/35 | CBT vs. IPT | --- | -0.37 [-0.83, 0.10] | | --- | --- | --- | 1.06 [0.02, 54.75] |
| McLean & Hakstian (1979) | 40/37 | BA vs. DYN | -0.73 [-1.17, -0.30] | --- | | --- | 3.00 [1.21, 7.47] | BDI ≤ 7 | 8.04 [1.64, 39.30] |
| McNamara & Horan (1986) | 12/12 | BA vs. CBT (according to Beck) | -0.25 [-1.13, 0.63] | --- | | --- | --- | --- | 1.00 [0.12, 8.56] |
|  | 12/13 | BA vs. SUP (referring to Rogers) | -0.84 [-1.76, 0.09] | --- | | --- | --- | --- | 1.50 [0.20, 11.00] |
|  | 12/13 | CBT (according to Beck) vs. SUP (referring to Rogers) | -0.60 [-1.51, 0.30] | --- | | --- | --- | --- | 1.50 [0.20, 11.00] |
| Milgrom et al. (2005) | 46/66 | BA vs. SUP | 0.27 [-0.11, 0.65] | --- | | --- | 0.63 [0.25, 1.61] | BDI < 17 | 0.46 [0.20, 1.04] |
|  | 46/47 | BA vs. SUP | -0.26 [-0.67, 0.15] | --- | | --- | 0.75 [0.26, 2.16] | BDI < 17 | 1.06 [0.46, 2.42] |
|  | 66/47 | SUP vs. SUP | -0.56 [-0.95, -0.18] | --- | | --- | 1.19 [0.46, 3.07] | BDI < 17 | 2.31 [1.02, 5.23] |
| Miller et al. (1989) | 15/14 | CBT (according to Beck) vs. Social skills training | 1.47 [0.62, 2.32] | 1.77 [0.87, 2.66] | | 1.62 [0.89, 2.35] | 4.00 [0.59, 27.25] | BDI ≤ 9 + HRSD < 7 | 0.33 [0.05, 2.10] |
| Mohr et al. (2001) | 20/22 | CBT vs. SUP | -0.57 [-1.21, 0.08] | -0.46 [-1.10, 0.18] | | -0.51 [-1.05, 0.02] | 3.33 [0.57, 19.42] | diagnosis of depression not present anymore | 4.22 [0.43, 41.45] |
| O'Leary & Beach (1990) | 15/15 | CBT (according to Beck) vs. Problem solving therapy | 0.60 [-0.22, 1.42] | --- | | --- | --- | --- | 1.00 [0.17, 5.98] |
| Power & Freeman (2012) | 46/54 | CBT (according to Beck) vs. IPT | 0.28 [-0.25, 0.80] | --- | | --- | --- | --- | 0.35 [0.15, 0.81] |
| Rude (1986) | 16/16 | CBT vs. Social skills training | -0.21 [-0.96, 0.53] | --- | | --- | --- | --- | 1.00 [0.12, 8.13] |
| Sanchez et al. (1980) | 17/17 | DYN vs. Social skills training | --- | --- | | --- | --- | --- | 1.00 [0.22, 4.46] |
| Schramm et al. (2011) | 14/15 | Cognitive behavioral analysis system of psychotherapy vs. IPT | -0.85 [-1.61, -0.08] | -0.66 [-1.41, 0.09] | | -0.75 [-1.39, -0.12] | 5.33 [1.02, 27.76] | HRSD ≤ 8 | 2.00 [0.16, 24.87] |
| Shapiro et al. (1994) | 38/37 | CBT (according to Beck) vs. DYN | -0.19 [-0.71, 0.33] | --- | | --- | --- | --- | 1.04 [0.36, 2.99] |
| Shaw (1977) | 8/8 | BA vs. CBT (according to Beck) | 1.13 [-0.05, 2.21] | 0.94 [-0.11, 1.99] | | 1.04 [0.15, 1.93] | 0.20 [0.02, 1.71] | BDI ≤ 9 | 1.00 [0.02, 56.86] |
| Steuer et al. (1984) | 16/17 | CBT (according to Beck) vs. DYN | -0.68 [-1.39, 0.02] | -0.20 [-0.88, 0.49] | | -0.44 [-1.02, 0.14] | 2.80 [0.56, 13.95] | HRSD ≤ 6 | 1.17 [0.29, 4.73] |
| Strauman et al. (2006) | 21/24 | CBT (according to Beck) vs. Self-system therapy | 0.32 [-0.27, 0.91] | 0.15 [-0.43, 0.74] | | 0.24 [-0.25, 0.73] | 0.56 [0.15, 2.10] | HRSD < 6 | 0.86 [0.15, 4.79] |
| Teri et al. (1997) | 23/19 | BA vs. Problem solving therapy | -0.53 [-1.15, 0.09] | -0.35 [-0.96, 0.26] | | -0.44 [-0.96, 0.07] | 0.50 [0.14, 1.79] | diagnosis of depression not present anymore | 1.21 [0.02, 63.94] |
| Thompson et al. (1987) | 34/41 | BA vs. CBT (according to Beck) | -0.13 [-0.63, 0.37] | -0.22 [-0.72, 0.29] | | -0.17 [-0.60, 0.25] | 2.53 [0.80, 7.98] | diagnosis of depression not present anymore | 2.42 [0.68, 8.56] |
|  | 34/34 | BA vs. DYN | -0.23 [-0.74, 0.27] | -0.17 [-0.68, 0.34] | | -0.20 [-0.63, 0.22] | 1.71 [0.52, 5.62] | diagnosis of depression not present anymore | 1.00 [0.23, 4.37] |
|  | 41/34 | CBT (according to Beck) vs. DYN | -0.07 [-0.57, 0.44] | 0.07 [-0.43, 0.57] | | 0.00 [-0.42, 0.42] | 0.68 [0.23, 1.97] | diagnosis of depression not present anymore | 0.41 [0.12, 1.46] |
| Watson et al. (2003) | 45/40 | CBT (according to Beck) vs. Process-experiential therapy | -0.04 [-0.47, 0.38] | --- | | --- | 0.78 [0.30, 2.07] | Reliable change index | 0.58 [0.20, 1.67] |
| Wilson et al. (1983) | 8/8 | BA vs. CBT (according to Beck) | -0.24 [-1.23, 0.74] | -0.14 [-1.12, 0.84] | | -0.19 [-1.01, 0.63] | --- | --- | 4.20 [0.33, 53.13] |
| Zettle & Rains (1989) | 12/13 | Acceptance and commitment therapy vs. CBT (according to Beck) | -0.52 [-1.39, 0.36] | -0.34 [-1.21, 0.52] | | -0.43 [-1.16, 0.30] | 0.75 [0.12, 4.66] | BDI ≤ 15 | 3.30 [0.29, 37.10] |

*Note. n/n* = Number of participants per treatment arm. BA = behavior activation therapy, CBT = cognitive behavior therapy, DYN = psychodynamic therapy, IPT = interpersonal therapy, SUP = supportive therapies. Numbers are Hedges’ *g* and 95% confidence intervals with regard to patient self-ratings, clinician ratings, and the combined outcome measure (negative effect sizes indicate that the first treatment was more efficacious than the second), and odds ratios and 95% confidence intervals with regard to clinical significance and drop-out (*OR* > 1 indicates that the odds of remission/completing were higher in the first treatment than in the second).
