# Supplementary material for: Comparing Bona Fide Psychotherapies of Depression in Adults with Two Meta-Analytical Approaches
Source: PLoS One. 2013 Jun 28;8(6):e68135. doi: 10.1371/journal.pone.0068135 (PMC3695954; doi:10.1371/journal.pone.0068135)
Supplement: Appendix S1 — Studies included in the meta-analyses. (DOCX) [file pone.0068135.s003.docx]

Appendix S1: Studies included in the meta-analyses

Alladin A, Alibhai A (2007) Cognitive hypnotherapy for depression: an empirical investigation. Int J Clin Exp Hypn 55: 147-166. doi:10.1080/00207140601177897

Arean PA, Raue P, Mackin RS, Kanellopoulos D, McCulloch C, et al. (2010) Problem-solving therapy and supportive therapy in older adults with major depression and executive dysfunction. Am J of Psychiatry 167, 1391-1398. doi:10.1176/appi.ajp.2010.09091327

Barkham M, Shapiro DA, Hardy GE, Rees A (1999) Psychotherapy in two-plus-one sessions: outcomes of a randomized controlled trial of cognitive-behavioral and psychodynamic-interpersonal therapy for subsyndromal depression. J Consult Clin Psychol 67, 201-211. doi:10.1037/0022-006x.67.2.201

Beutler LE, Engle D, Mohr D, Daldrup RJ, Bergan J, et al. (1991) Predictors of differential response to cognitive, experiential, and self-directed psychotherapeutic procedures. J Consult Clin Psychol 59: 333-340. doi:10.1037//0022-006x.59.2.333

Beutler LE, Moleiro C, Malik M, Harwood T M, Romanelli R, et al. (2003) A comparison of the dodo, EST, and ATI factors among comorbid stimulant dependent, depressed patients. Clin Psychol Psychother 10: 69-85. doi: 10.1002/cpp.354

Bodenmann G, Plancherel B, Beach SRH, Widmer K, Gabriel B, et al. (2008) Effects of coping-oriented couples therapy on depression: a randomized clinical trial. J Consult Clin Psychol 76: 944-954. doi:10.1037/a0013467

Bright JI, Baker KD, Neimeyer RA (1999) Professional and paraprofessional group treatments for depression: a comparison of cognitive-behavioral and mutual support interventions. J Consult Clin Psychol 67: 491-501. doi:10.1037/0022-006x.67.4.491

Comas-Díaz L (1981) Effects of cognitive and behavioral group treatment on the depressive symptomatology of puerto-rican women. J Consult Clin Psychol 49: 627-632. doi:10.1037/0022-006x.49.5.627

Cooper PJ, Murray L, Wilson A, Romaniuk H (2003) Controlled trial of the short- and long-term effect of psychological treatment of post-partum depression: 1. Impact on maternal mood. Br J Psychiatry 182: 412-419. doi:10.1192/bjp.182.5.412

David D, Szentagotai A, Lupu V, Cosman D (2008) Rational emotive behavior therapy, cognitive therapy, and medication in the treatment of major depressive disorder: a randomized clinical trial, posttreatment outcomes, and six-month follow-up. J Clin Psychol 64: 728-746. doi:10.1002/jclp.20487

Dimidjian S, Hollon SD, Dobson KS, Schmaling KB, Kohlenberg RJ, et al. (2006) Randomized trial of behavioral activation, cognitive therapy, and antidepressant medication in the acute treatment of adults with major depression. J Consult Clin Psychol 74: 658-670. doi:10.1037/0022-006x.74.4.658

Elkin I, Shea MT, Watkins JT, Imber SD, Sotsky SM, et al. (1989) Treatment of depression collaborative research program: general effectiveness of treatments. Arch Gen Psychiatry 46: 971-982. doi:10.1001/archpsyc.1989.01810110013002

Evans RL, Connis RT (1995) Comparison of brief group therapies for depressed cancer-patients receiving radiation treatment. Public Health Rep 110: 306-311.

Forman EM, Herbert JD, Moitra E, Yeomans PD, Geller PA (2007) A randomized controlled effectiveness trial of acceptance and commitment therapy and cognitive therapy for anxiety and depression. Behav Modif 31: 772-799. doi:10.1177/0145445507302202

Gallagher DE, Thompson LW (1982) Treatment of major depressive disorder in older adult outpatients with brief psychotherapies. Psychotherapy-Theory Research and Practice 19: 482-490. doi:10.1037/h0088461

Gallagher-Thompson D, Steffen AM (1994) Comparative effects of cognitive-behavioral and brief psychodynamic psychotherapies for depressed family caregivers. J Consult Clin Psychol 62: 543-549. doi:10.1037/0022-006x.62.3.543

Goldman RN, Greenberg LS, Angus L (2006) The effects of adding emotion-focused interventions to the client-centered relationship conditions in the treatment of depression. Psychother Res 16: 536-546. doi:10.1080/10503300600589456

Greenberg LS, Watson J (1998) Experiential therapy of depression: differential effects of client-centered relationship conditions and process experiential interventions. Psychother Res 8: 210-224. doi:10.1093/ptr/8.2.210

Hersen M, Bellack AS, Himmelhoch JM, Thase ME (1984) Effects of social skill training, amitriptyline, and psychotherapy in unipolar depressed women. Behav Ther 15: 21-40. doi:10.1016/s0005-7894(84)80039-8

Hogg JA, Deffenbacher JL (1988) A comparison of cognitive and interpersonal process group therapies in the treatment of depression among college students. J Couns Psychol 35: 304-310. doi:10.1037//0022-0167.35.3.304

Hopko DR, Armento MEA, Robertson SMC, Ryba MM, Carvalho JP, et al. (2011) Brief behavioral activation and problem-solving therapy for depressed breast cancer patients: randomized trial. J Consult Clin Psychol 79: 834-849. doi:10.1037/a0025450

Jacobson NS, Fruzzetti AE, Dobson K, Schmaling KB, Salusky S (1991) Marital-therapy as a treatment for depression. J Consult Clin Psychol 59: 547-557. doi:10.1037/0022-006x.59.4.547

Kelly JA, Murphy DA, Bahr GR, Kalichman SC, Morgan MG, et al. (1993) Outcome of cognitive-behavioral and support group brief therapies for depressed, HIV-infected persons*.* Am J Psychiatry 150: 1679-1686.

King M, Sibbald B, Ward E, Bower P, Lloyd M, et al. (2000) Randomized controlled trial of non-directive counselling, cognitive-behaviour therapy and usual general practitioner care for patients with depression. I: clinical effectiveness. BMJ 321: 1383-1388. doi:10.1136/bmj.321.7273.1383

Kiosses DN, Arean PA, Teri L, Alexopoulos GS (2010) Home-delivered problem adaptation therapy (PATH) for depressed, cognitively impaired, disabled elders: a preliminary study. Am J Geriatr Psychiatry 18: 988-998. doi:10.1097/JGP.0b013e3181d6947d

Kocsis JH, Gelenberg AJ, Rothbaum BO, Klein DN, Trivedi MH, et al. (2009) Cognitive behavioral analysis system of psychotherapy and brief supportive psychotherapy for augmentation of antidepressant nonresponse in chronic depression. Arch Gen Psychiatry 66: 1178-1188.

Kornblith SJ, Rehm LP, Ohara MW, Lamparski DM (1983) The contribution of self-reinforcement training and behavioral assignments to the efficacy of self-control therapy for depression. Cognit Ther Res 7: 499-527. doi:10.1007/bf01172888

Koszycki D, Bisserbe JC, Blier P, Bradwejn J, Markowitz J (2012) Interpersonal psychotherapy versus brief supportive therapy for depressed infertile women: first pilot randomized controlled trial. Arch Womens Ment Health 15: 193-201. doi:10.1007/s00737-012-0277-z

Luty SE, Carter JD, McKenzie JM, Rae AM, Frampton CMA, et al. (2007) Randomised controlled trial of interpersonal psychotherapy and cognitive-behavioural therapy for depression. Br J Psychiatry 190: 496-502. doi:10.1192/bjp.bp.106.024729

Maina G, Forner F, Bogetto F (2005) Randomized controlled trial comparing brief dynamic and supportive therapy with waiting list condition in minor depressive disorders. Psychother Psychosom 74: 43-50. doi:10.1159/000082026

Manicavasgar V, Parker G, Perich T (2011) Mindfulness-based cognitive therapy vs cognitive behaviour therapy as a treatment for non-melancholic depression. J Affect Disord 130: 138-144. doi:10.1016/j.jad.2010.09.027

Markowitz JC, Kocsis JH, Bleiberg KL, Christos PJ, Sacks M (2005) A comparative trial of psychotherapy and pharmacotherapy for "pure" dysthymic patients. J Affect Disord 89: 167-175. doi:10.1016/j.jad.2005.10.001

Markowitz JC, Kocsis JH, Fishman B, Spielman LA, Jacobsberg LB, et al. (1998) Treatment of depressive symptoms in human immunodeficiency virus-positive patients. Arch Gen Psychiatry 55: 452-457. doi:10.1001/archpsyc.55.5.452

Marshall MB, Zuroff DC, McBride C, Bagby RM (2008) Self-criticism predicts differential response to treatment for major depression. J Clin Psychol 64: 231-244. doi:10.1002/jclp.20438

McLean PD, Hakstian AR (1979) Clinical depression: comparative efficacy of outpatient treatments*.* Journal Consult Clin Psychol 47: 818-836. doi:10.1037//0022-006x.47.5.818

McNamara K, Horan JJ (1986) Experimental construct validity in the evaluation of cognitive and behavioral treatments for depression. J Couns Psychol 33: 23-30. doi:10.1037//0022-0167.33.1.23

Milgrom J, Negri LM, Gemmill AW, McNeil M, Martin PR (2005) A randomized controlled trial of psychological interventions for postnatal depression. Br J Clin Psychol 44: 529-542. doi:10.1348/014466505x34200

Miller IW, Norman WH, Keitner GI, Bishop SB, Dow MG (1989) Cognitive-behavioral treatment of depressed inpatients. Behav Ther 20: 25-47. doi:10.1016/s0005-7894(89)80116-9

Mohr DC, Boudewyn AC, Goodkin DE, Bostrom A, Epstein L (2001) Comparative outcomes for individual cognitive-behavior therapy, supportive-expressive group psychotherapy, and sertraline for the treatment of depression in multiple sclerosis. J Consult Clin Psychol 69: 942-949. doi:10.1037/0022-006x.69.6.942

O'Leary KD, Beach SRH (1990) Marital-therapy: a viable treatment for depression and marital discord. Am J Psychiatry 147: 183-186.

Power MJ, Freeman C (2012) A randomized controlled trial of IPT versus CBT in primary care: with some cautionary notes about handling missing values in clinical trials. Clinical Psychol Psychother 19: 159-169. doi:10.1002/cpp.1781

Rude SS (1986) Relative benefits of assertion or cognitive self-control treatment for depression as a function of proficiency in each domain. J Consult Clin Psychol 54: 390-394. doi:10.1037//0022-006x.54.3.390

Sanchez VC, Lewinsohn PM, Larson DW (1980) Assertion training: effectiveness in the treatment of depression. J Clin Psychol 36: 526-529.

Schramm E, Zobel I, Dykierek P, Kech S, Brakemeier EL, et al. (2011) Cognitive behavioral analysis system of psychotherapy versus interpersonal psychotherapy for early-onset chronic depression: a randomized pilot study. J Affect Disord 129: 109-116. doi:10.1016/j.jad.2010.08.003

Shapiro DA, Barkham M, Rees A, Hardy GE, Reynolds S, et al. (1994) Effects of treatment duration and severity of depression on the effectiveness of cognitive-behavioral and psychodynamic-interpersonal psychotherapy. J Consult Clin Psychol 62: 522-534. doi:10.1037/0022-006x.62.3.522

Shaw BF (1977) Comparison of cognitive therapy and behavior therapy in the treatment of depression. J Consult Clin Psychol 45: 543-551. doi:10.1037/0022-006x.45.4.543

Steuer JL, Mintz J, Hammen CL, Hill MA, Jarvik LF, et al. (1984) Cognitive-behavioral and psychodynamic group-psychotherapy in treatment of geriatric depression. J Consult Clin Psychol 52: 180-189. doi:10.1037//0022-006x.52.2.180

Strauman TJ, Vieth AZ, Merrill KA, Kolden GG, Woods TE, et al. (2006) Self-system therapy as an intervention for self-regulatory dysfunction in depression: a randomized comparison with cognitive therapy. J Consult Clin Psychol 74: 367-376. doi:10.1037/0022-006x.74.2.367

Teri L, Logsdon RG, Uomoto J, McCurry SM (1997) Behavioral treatment of depression in dementia patients: a controlled clinical trial. J Gerontol B Psychol Sci Soc Sci 52: 159-166. doi: 10.1093/geronb/52B.4.P159

Thompson LW, Gallagher D, Breckenridge JS (1987) Comparative effectiveness of psychotherapies for depressed elders. J Consult Clin Psychol 55: 385-390. doi:10.1037//0022-006x.55.3.385

Watson JC, Gordon LB, Stermac L, Kalogerakos F, Steckley P (2003) Comparing the effectiveness of process-experiential with cognitive-behavioral psychotherapy in the treatment of depression. J Consult Clin Psychol 71: 773-781. doi:10.1037/0022-006x.71.4.773

Wilson PH, Goldin JC, Charbonneaupowis M (1983) Comparative efficacy of behavioral and cognitive treatments of depression. Cognit Ther Res 7: 111-124. doi:10.1007/bf01190064

Zettle RD, Rains JC (1989) Group cognitive and contextual therapies in treatment of depression. J Clin Psychol 45: 436-445. doi:10.1002/1097-4679(198905)45:3<436::aid-jclp2270450314>3.0.co;2-l
