# Supplementary material for: Comparing Bona Fide Psychotherapies of Depression in Adults with Two Meta-Analytical Approaches
Source: PLoS One. 2013 Jun 28;8(6):e68135. doi: 10.1371/journal.pone.0068135 (PMC3695954; doi:10.1371/journal.pone.0068135)
Supplement: Appendix S2 — Studies that were excluded in the last step. (DOCX) [file pone.0068135.s004.docx]

**Appendix S2: Studies that were excluded in the last step**

Studies, where less than two treatments were bona fide

Alexopoulos GS, Raue P, Arean P (2003) Problem-solving therapy versus supportive therapy in geriatric major depression with executive dysfunction. Am J Geriatr Psychiatry 11: 46-52. doi: 10.1176/appi.ajgp.11.1.46

Arean PA, Perri MG, Nezu AM, Schein RL, Christopher F, et al. (1993) Comparative effectiveness of social problem-solving therapy and reminiscence therapy as treatments for depression in older adults. J Consult Clin Psychol 61: 1003-1010. doi: 10.1037/0022-006x.61.6.1003

Ayen I, Hautzinger M (2004) Cognitive behavior therapy for depression in menopausal women. A controlled, randomized treatment study. Z Klin Psychol Psychother 33: 290-299. doi: 10.1026/1616-3443.33.4.290

Baker AL, Kavanagh DJ, Kay-Lambkin FJ, Hunt SA, Lewin TJ, et al. (2010) Randomized controlled trial of cognitive-behavioural therapy for coexisting depression and alcohol problems: short-term outcome. Addiction 105: 87-99. doi: 10.1111/j.1360-0443.2009.02757.x

Bowman D, Scogin F, Lyrene B (1995) The efficacy of self-examination therapy and cognitive bibliotherapy in the treatment of mild to moderate depression. Psychother Res 5: 131-140. doi: 10.1080/10503309512331331256

Constantino MJ, Marnell ME, Haile AJ, Kanther-Sista SN, Wolman K, et al. (2008) Integrative cognitive therapy for depression: a randomized pilot comparison. Psychotherapy 45: 122-134. doi: 10.1037/0033-3204.45.2.122

Covi L, Lipman RS (1987) Cognitive behavioral group-psychotherapy combined with imipramine in major depression. Psychopharmacol Bull 23: 173-176.

Dowrick C, Dunn G, Ayuso-Mateos JL, Dalgard OS, Page H, et al. (2000). Problem solving treatment and group psychoeducation for depression: multicentre randomised controlled trial. Br Med J 321: 1450-1454. doi: 10.1136/bmj.321.7274.1450

Emanuels-Zuurveen L, Emmelkamp PMG (1996) Individual behavioural-cognitive therapy v. marital therapy for depression in maritally distressed couples. Br J Psychiatry 169: 181-188. doi: 10.1192/bjp.169.2.181

Fleming BM, Thornton DW (1980) Coping skills training as a component in the short-term treatment of depression. J Consult Clin Psychol 48: 652-654. doi: 10.1037//0022-006x.48.5.652

Foster RP (2007) Treating depression in vulnerable urban women: a feasibility study of clinical outcomes in community service settings. Am J Orthopsychiatry 77: 443-453. doi: 10.1037/0002-9432.77.3.443

Freedland KE, Skala JA, Carney RM, Rubin EH, Lustman PJ, et al. (2009) Treatment of depression after coronary artery bypass surgery: a randomized controlled trial. Arch Gen Psychiatry 66: 387-396.

Fuchs CZ, Rehm LP (1977) A self-control behavior-therapy program for depression. J Consult Clin Psychol 45: 206-215. doi: 10.1037/0022-006x.45.2.206

Gallagher D (1981) Behavioral group therapy with elderly depressives: An experimental study. In Upper D, Ross S, editors. Behavioral group therapy. Champaign, IL: Research Press. pp. 187-224.

Gardner P, Oei TPS (1981) Depression and self-esteem: an investigation that used behavioral and cognitive approaches to the treatment of clinically depressed clients. J Clin Psychol 37: 128-135. doi: 10.1002/1097-4679(198101)37:1<128::aid-jclp2270370124>3.0.co;2-1

Hautzinger M, Welz S (2008) Short- and long-term efficacy of psychological intervention for depression in older adults. Z Klin Psychol Psychother 37: 52-60. doi: 10.1026/1616-3443.37.1.52

Hopko DR, Lejuez CW, LePage JP, Hopko SD, McNeil DW (2003) A brief behavioral activation treatment for depression - a randomized pilot trial within an inpatient psychiatric hospital. Behav Modif 27: 458-469. doi: 10.1177/0145445503255489

Hussian RA, Lawrence PS (1981) Social reinforcement of activity and problem-solving training in the treatment of depressed institutionalized elderly patients. Cognit Ther Res 5: 57-69. doi: 10.1007/bf01172326

Jacobson NS, Dobson KS, Truax PA, Addis ME, Koerner K, et al. (1996). A component analysis of cognitive-behavioral treatment for depression. J Consult Clin Psychol 64: 295-304. doi: 10.1037//0022-006x.64.2.295

LaPointe KA, Rimm DC (1980) Cognitive, assertive, and insight-oriented group therapies in the treatment of reactive depression in women. Psychotherapy – Theory Research and Practice 17: 312-321. doi: 10.1037/h0085928

Linde JA, Simon GE, Ludman EJ, Ichikawa LE, Operskalski BH, et al. (2011) A randomized controlled trial of behavioral weight loss treatment versus combined weight loss/depression treatment among women with comorbid obesity and depression. Ann Behav Med 41: 119-130. doi: 10.1007/s12160-010-9232-2

Lynch D, Tamburrino M, Nagel R, Smith MK (2004) Telephone-based treatment for family practice patients with mild depression. Psychol Rep 94: 785-792. doi: 10.2466/pr0.94.3.785-792

Markowitz JC, Kocsis JH, Christos P, Bleiberg K, Carlin A (2008) Pilot study of interpersonal psychotherapy versus supportive psychotherapy for dysthymic patients with secondary alcohol abuse or dependence. J Nerv Ment Dis 196: 468-474. doi: 10.1097/NMD.0b013e31817738f1

Murphy GE, Carney RM, Knesevich MA, Wetzel RD, Whitworth P (1995) Cognitive behavior therapy, relaxation training, and tricyclic antidepressant medication in the treatment of depression. Psychol Rep 77: 403-420.

Nezu AM (1986) Efficacy of social problem-solving therapy approach for unipolar depression. J Consult Clin Psychol 54: 196-202. doi: 10.1037//0022-006x.54.2.196

Padfield M (1976) The comparative effects of two counseling approaches on intensity of depression among rural women of low socioeconomic status. J Couns Psychol 23: 209-214. doi: 10.1037/0022-0167.23.3.209

Reynolds CF, Dew MA, Martire LM, Miller MD, Cyranowski JM, et al. (2010) Treating depression to remission in older adults: a controlled evaluation of combined escitalopram with interpersonal psychotherapy versus escitalopram with depression care management. Int J Geriatr Psychiatry 25: 1134-1141. doi: 10.1002/gps.2443

Scott AIF, Freeman CPL (1992) Edinburgh primary care depression study - treatment outcome, patient satisfaction, and cost after 16 weeks. Br Med J 304: 883-887.

Taylor FG, Marshall WL (1977) Experimental analysis of a cognitive-behavioral therapy for depression. Cognit Ther Res 1: 59-72.

Verduyn C, Barrowclough C, Roberts J, Tarrier N, Harrington R (2003) Maternal depression and child behaviour problems. Randomised placebo-controlled trial of a cognitive-behavioural group intervention. Br J Psychiatry 183: 342-348. doi: 10.1192/bjp.183.4.342

Vitriol VG, Ballesteros ST, Florenzano RU, Weil KP, Benadof DF (2009) Evaluation of an outpatient intervention for women with severe depression and a history of childhood trauma. Psychiatr Serv 60: 936-942.

Watkins ER, Baeyens CB, Read R (2009) Concreteness training reduces dysphoria: proof-of-principle for repeated cognitive bias modification in depression. J Abnorm Psychol 118: 55-64. doi: 10.1037/a0013642

Wilson PH (1982) Combined pharmacological and behavioral treatment of depression. Behav Res Ther 20: 173-184. doi: 10.1016/0005-7967(82)90116-4

Studies, where effect sizes could not be calculated

Barkham M, Rees A, Stiles WB, Agnew RM, Halstead J, et al. (1996) Outcomes of time-limited psychotherapy in applied settings: replicating the Second Sheffield Psychotherapy Project. J Consult Clin Psychol 64: 1079-1085. doi: 10.1037/0022-006x.64.5.1079

Heckman TG, Sikkema KJ, Hansen N, Kochman A, Heh V, Neufeld S (2011) A randomized clinical trial of a coping improvement group intervention for HIV-infected older adults. Journal of Behavior Medicine 34: 102-111. doi: 10.1007/s10865-010-9292-6

Neimeyer RA, Weiss ME (1990) Cognitive and symptomatic predictors of outcome of group therapies for depression. J Cogn Psychother 4: 23-32.

Zeiss AM, Lewinsohn PM, Munoz RF (1979) Nonspedific improvement effects in depression using interpersonal skills training, pleasant activity schedules or cognitive training. J Consult Clin Psychol 47: 427-439. doi: 10.1037/0022-006x.47.3.427
