# Supplementary material for: Comparing Bona Fide Psychotherapies of Depression in Adults with Two Meta-Analytical Approaches
Source: PLoS One. 2013 Jun 28;8(6):e68135. doi: 10.1371/journal.pone.0068135 (PMC3695954; doi:10.1371/journal.pone.0068135)
Supplement: Appendix S3 — WinBUGS code used for mixed treatment comparisons. (DOCX) [file pone.0068135.s005.docx]

**Appendix S3: WinBUGS code used for mixed treatment comparisons**

**WinBUGS code used for clinical significance**

model{

sw[1]<-0

for(i in 1:70) {

logit(p[i])<-mu[s[i]]+ delta[i] * (1-equals(t[i],b[i]))

r[i]~dbin(p[i],n[i])

rh[i]<-n[i]*p[i]

dev[i] <- 2* (r[i] * (log(r[i]) - log(rh[i])) + (n[i]-r[i])*(log(n[i]-r[i]) - log(n[i]-rh[i])))

delta[i] ~ dnorm(md[i], tau)

taud[i] <- tau*(1+equals(m[i],3)/3)

md[i] <- d[t[i]] - d[b[i]] + equals(m[i],3) * sw[i] }

for (i in 2:70) { sw[i] <- (delta[i-1] - d[t[i-1]] + d[b[i-1]])/2}

for(j in 1:33) { mu[j]~dnorm(0,.0001) }

d[1]<-0

for (k in 2:13) {d[k] ~ dnorm(0,.001) }

sd<-1/sqrt(tau)

tau~dgamma(0.001,0.001)

for (k in 1:13) { rk[k]<- 13+1 - rank(d[],k)

best[k]<-equals(rk[k],1) }

sumdev <- sum(dev[])

}

**WinBUGS code used for continuous data (i.e., self-ratings and clinician ratings)**

model{

for(i in 1:ns2) {

y[i,2] ~ dnorm(delta[i,2],prec[i,2])

resdev[i] <- (y[i,2]-delta[i,2])*(y[i,2]-delta[i,2])*prec[i,2]

}

for(i in (ns2+1):(ns2+ns3)) {

for (k in 1:(na[i]-1)) {

for (j in 1:(na[i]-1)) {

Sigma[i,j,k] <- V[i]*(1-equals(j,k)) + var[i,k+1]*equals(j,k)

}

}

Omega[i,1:(na[i]-1),1:(na[i]-1)] <- inverse(Sigma[i,,])

y[i,2:na[i]] ~ dmnorm(delta[i,2:na[i]],Omega[i,1:(na[i]-1),1:(na[i]-1)])

for (k in 1:(na[i]-1)){

ydiff[i,k]<- y[i,(k+1)] - delta[i,(k+1)]

z[i,k]<- inprod2(Omega[i,k,1:(na[i]-1)], ydiff[i,1:(na[i]-1)])

}

resdev[i]<- inprod2(ydiff[i,1:(na[i]-1)], z[i,1:(na[i]-1)])

}

for(i in 1:(ns2+ns3)){

w[i,1] <- 0

delta[i,1] <- 0

for (k in 2:na[i]) {

var[i,k] <- pow(se[i,k],2)

prec[i,k] <- 1/var[i,k]

}

for (k in 2:na[i]) {

delta[i,k] ~ dnorm(md[i,k],taud[i,k])

md[i,k] <- d[t[i,k]] - d[t[i,1]] + sw[i,k]

taud[i,k] <- tau *2*(k-1)/k

w[i,k] <- (delta[i,k] - d[t[i,k]] + d[t[i,1]])

sw[i,k] <- sum(w[i,1:k-1])/(k-1)

}

}

totresdev <- sum(resdev[])

d[1]<-0

for (k in 2:nt){ d[k] ~ dnorm(0,.0001) }

sd ~ dunif(0,5)

tau <- pow(sd,-2)

for (k in 1:nt) {

rk[k] <- nt+1-rank(d[],k)

best[k] <- equals(rk[k],1) }

}
